# Detecting depression severity using weighted random forest and oxidative stress biomarkers

**DOI:** 10.1038/s41598-024-67251-y

**Published:** 2024-07-15

**Authors:** Mariam Bader, Moustafa Abdelwanis, Maher Maalouf, Herbert F. Jelinek

**Affiliations:** 1 Department of Management Science and Engineering, Khalifa University of Science and Technology, P.O. Box 127788, Abu Dhabi, United Arab Emirates; 2 Department of Medical Science, Biotechnology Center, Khalifa University of Science and Technology, P.O. Box 127788, Abu Dhabi, United Arab Emirates

**Keywords:** Depression severity, PHQ-9, Biochemistry, Machine learning, Oxidative stress, Bioinformatics, Computational biology and bioinformatics, Data mining, Machine learning, Predictive medicine, Diagnostic markers, Predictive markers, Depression

## Abstract

This study employs machine learning to detect the severity of major depressive disorder (MDD) through binary and multiclass classifications. We compared models that used only biomarkers of oxidative stress with those that incorporate sociodemographic and health-related factors. Data collected from 830 participants, based on the Patient Health Questionnaire (PHQ-9) score, inform our analysis. In binary classification, the Random Forest (RF) classifier achieved the highest Area Under the Curve (AUC) of 0.84 when all features were included. In multiclass classification, the AUC improved from 0.84 with only oxidative stress biomarkers to 0.88 when all characteristics were included. To address data imbalance, weighted classifiers, and Synthetic Minority Over-sampling Technique (SMOTE) approaches were applied. Weighted random forest (WRF) improved multiclass classification, achieving an AUC of 0.91. Statistical tests, including the Friedman test and the Conover post-hoc test, confirmed significant differences between model performances, with WRF using all features outperforming others. Feature importance analysis shows that oxidative stress biomarkers, particularly GSH, are top ranked among all features. Clinicians can leverage the results of this study to improve their decision-making processes by incorporating oxidative stress biomarkers in addition to the standard criteria for depression diagnosis.

## Introduction

Depression affects a large part of the population around the world and is expected to increase substantially due to several factors such as evolutionary mismatch between past and modern environments, declining social connections and increased loneliness^1^. Globally, approximately 280 million people suffer from depression, with an estimated 5% among adults and 5.7% among older people^2^. According to the World Health Organization^3^, depression is considered a significant contributor to the burden of disease and an important cause of disability. It can also cause severe socioeconomic problems and a long-term impact on the economy^4,5^. Current methods for diagnosing depression focus primarily on responses and behavioral activities and the use of questionnaires such as the Primary Health Questionnaire and the Beck Depression Inventory^6^. Previous studies on depression have investigated several tools, techniques, and factors for detecting depression with mixed results. Some of the reported inconsistencies are probably related to methodological differences in sample size, study design, and analytical tools^7,8^. Consequently, research continues to focus on the timely detection of depression using the primary association between depression and biological, clinical, and sociodemographic factors.

Risk assessments of depression and severity are complex and require a detailed investigation to determine dominant factors associated with depression, including genetic predisposition, comorbidities, and physiological factors such as oxidative stress and inflammation^9–11^. Oxidative stress is an imbalance between excess production of reactive oxygen species (ROS) and antioxidants that manifest themselves in changes in biomarkers of oxidative stress, including 8-isoprostane (8-iso-PGF2$\alpha$), 8-hydroxy-2’-deoxyguanosine (8-OHdG) and the ubiquitous glutathione redox reaction (GSH-GSSG)^12^. There is evidence that suggests a positive correlation between 8-iso-PGF2$\alpha$, oxidized GSH (GSSG), and 8-OHdG and depression. GSH and the GSH-GSSG ratio are negatively associated with depression^13–15^. Furthermore, some studies have associated oxidative stress with angiotensin-converting enzyme (ACE) and treatment outcome^16–18^. However, the role of the ACE gene polymorphism and the connection between depression and oxidative stress have not been extensively studied, although there is a link between oxidative stress and depression^19–21^. Furthermore, depression is also associated with cardiovascular disease, diabetes, and hypertension^7,22,23^ and other factors, such as gender and age^24^.

Glutathione, 8-isoprostane, and 8-hydroxy-2’-deoxyguanosine (8-OHdG)), are among the most investigated oxidative stress biomarkers for depression^25–28^. However, the combined role of these biomarkers in the classification of depression and progression has not been investigated^29,30^. Our current study uses machine learning models to detect depression, comparing the effectiveness of oxidative stress biomarkers alone, clinical and demographic markers, and their combination. This approach fills a gap in previous research, which mainly focused on sociodemographic characteristics^19,31^.In addition, our study addresses not only depression but also the progression of depression (four severity levels defined by PHQ-9), which has not been shown previously.

The paper is structured as follows: “Related works” reviews related work. “Data and participants” details the process of collecting and preprocessing the data set, while “Methods” outlines the methodology used. The results are presented in “Results”, followed by a discussion of our findings, limitations, and potential scopes of future research in “Discussion”. Finally, “Conclusion” presents the conclusions.

## Related works

The literature shows that depression could be associated with chronic diseases. Hooker et al.^32^ performed a cross-sectional study using the patient health questionnaire (PHQ) and demonstrated a significant association between depressed people and cardiovascular disease. In this context, identifying associations between comorbidities, depression, and physiological markers requires the inclusion of medication use, as some antidepressants act as antioxidants as well^8^.

However, there is no consensus on how demographic factors, such as gender and age, contribute to depression. For example, a study by Zheng et al.^24^ reported that depression in women is higher than in men. This finding was also supported by a cross-sectional study by Li et al.^33^. Interestingly, another study by Kodydková^34^ showed that the concentration of GSH was higher in depressed women, supporting the association of gender and oxidative stress with depression. In contradiction, Cabello et al.^35^ found that young men are more likely to have depression than women, while Wicke et al.^13^ found no significant association between gender and depression. Whether these factors could act together in the progression of depression has not been investigated.

Many researchers have applied different machine learning methods to predict depression. Logistic Regression (LR), RF, K-Nearest Neighbors (KNN), Support Vector Machine (SVM), Naïve Bayes (NB), and Artificial Neural Networks (ANN) are among the most widely used machine learning algorithms^14–18^. Machine learning models were evaluated based on several performance metrics such as precision, precision, sensitivity, recall, and F1 score, particularly for unbalanced data. Sociodemographic data and results from the Beck Depression Inventory standardized (Standardized BDI) were used as input for an ANN approach by Cvetkovic^19^ to predict the depression range among 84 breast cancer patients. The results showed that demographic factors such as age, gender, education, marital status, occupation status, and economic status contribute to the detection of depression and can predict the severity of depression. Another study by Sau and Bhakta^20^ applied ten machine learning classifiers to diagnose depression among 520 older people according to sociodemographic and health-related factors. The highest predicted accuracy was 0.89 and 0.94 Area Under the Curve (AUC), which was achieved by RF. Priya et al.^21^ applied Decision Tree (DT), RF, NB, SVM, and KNN, to predict five levels of depression according to the DASS-21 questionnaire. Their findings revealed that RF had the best accuracy with imbalanced data.

Interestingly, Shen et al.^36^ used ANN and NB models to distinguish between bipolar disorder, schizophrenia, and MDD using five candidate genes (LYPD1, HMBS, HEBP2, SETD3, and ECM2). The results obtained from the ANN network showed that these candidate genes could perfectly distinguish bipolar disorder, schizophrenia, and MDD (0.77 and 0.82, respectively). Furthermore, Richter et al.^30^ employed machine learning to identify cognitive biases in subclinical anxious and depressed individuals, obtaining 0.71 accuracy for symptomatic individuals and 0.70 for controls. The findings reveal specific behavioral measures and highlight key cognitive mechanisms.

However, class imbalance is a common issue for machine learning classifiers, which greatly affects prediction performance. Zulfiker et al.^37^ used the SMOTE technique to overcome this obstacle. In their study, six machine learning models were applied to classify the study participants as depressed or did not use demographic and psychosocial information. The results indicated that the AdaBoost classifier provided the best classification with 0.92 accuracy and 0.96 AUC. An RF classifier combined with SMOTE also provided good results in predicting depression using the Korea Welfare Panel Study (KoWePS)^31^. Furthermore, this technique has been implemented to handle the imbalance class in a study conducted by Nandanwar and Nallamolu^38^ in which the AdaBoost Classifier showed the best performance with an F1 score of 0.93. SMOTE is based on the generation of new samples among minority classes without any change in the majority to balance the distribution of classes that could affect the original data used^39^. Taking into account its effectiveness in other studies, another approach is proposed to solve the problem of imbalanced data using the weighted class^40^. The idea of a weighted class is to penalize misclassification rather than to generate more samples^41^.

## Data and participants

The data used in this study were collected from the DiabHealh Center, Albury, Australia^42^. The data set included 2,621 observations from patients collected between 2002 and 2015. It consisted of 43.1% men and 56.9% women with a mean age of 66 years. To address multiple visits from the same patient, the data rows were filtered based on the most recent visit, resulting in 830 patients for the analysis. The study was carried out with the approval of the institutional Human Ethics Committee (ethics approval number: 2006/042), in strict adherence to the Declaration of Helsinki, ensuring that all participants provided their written informed consent. The level of depression was determined using the PHQ-9 and ranged from 0 to 24. Patients whose depression score was 4 or less were considered not depressed. A range of 4 to 13, indicated mild depression, 14 to 20 moderate depression, and a score of 21 or more was classified as severe depression^43^. The data set contains not only sociodemographic details such as age and gender, but also oxidative stress biomarkers, health-related factors, including medications for diabetes, hypertension, and cardiovascular diseases, and information on the haplotype of the angiotensin-converting enzyme (ACE) gene. There are 14 independent variables and one target variable is the PHQ score. Possible values/ranges, type of variable, and description of each variable are presented in Table 1.

**Table 1:** Variables used for depression detection.

Variable name	Variable description	Possible values/ranges
ACE	Angiotensin converting enzyme haplotype	1, 2, 3
8-u-isoprostane	8-u-Isoprostane	0.06–84.21
8-OHdG	8-Hydroxydeoxyguanosine	3.08–850.02 pg/ml
GSH	Glutathione	317.25–4336.47 mg/100 ml
GSSG	Oxidized glutathione	37.37–1518.43 mg/100 ml
GSH-GSSG-r	Oxidized glutathione:glutathione ratio	0.72–22.55
Depression Med	Whether the participant takes antidepression medication	Yes, No
DM-Status	Whether the participant has diabetes	Yes , No
DM-MedUse	Whether the participant takes diabetes medication	Yes , No
HT-Status	Whether the participant is suffering from hypertension	Yes , No
HT-MedUse	Whether the participant takes hypertension medication	Yes , No
CVD	Whether the participant is suffering from cardiovascular illness	Yes, No
Age	Age range of the participant	25–96 years
Gender	Gender of the participant	Male, Female
Depression	The patient health questionnaire score (PHQ-score)	0–24

**Figure 1:**
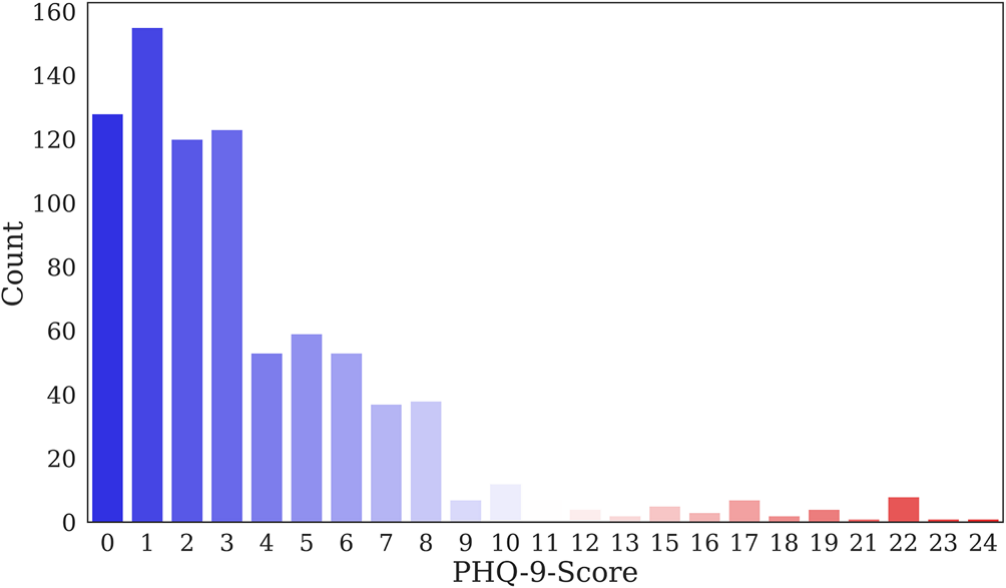
Distribution of depression levels of participants in the data set.

Figure 1 shows the distribution of depression levels between participants in the data set. Among the 830 participants, the PHQ-9 scores indicated that 579 had no depression, 219 had mild depression, 21 had a moderate level of depression, and 11 had severe depression.

Table 2 shows the distribution of depressed and non-depressed participants according to different criteria. For the binary model, participants with a PHQ score of more than four are considered to have depression regardless of its severity. Female participants were found to be more depressed than male participants.

**Table 2:** Distribution of depressed and not-depressed participants according to different criteria.

Criteria	Category	Total	Depressed	Non-depressed
Gender	Male	358	96	262
	Female	472	155	317
CVD	Yes	390	103	287
	No	440	148	292
DM-Status	Yes	243	127	116
	No	587	124	463
DM-MedUse	Yes	178	93	85
	No	652	158	494
HT-Status	Yes	421	134	287
	No	409	117	292
HT-MedUse	Yes	386	123	263
	No	444	128	316
Depression MedUse	Yes	107	42	65
	No	723	209	514

The percentage of depression among women was 32.8%, while 26.8% among men. The prevalence of depression in participants without cardiovascular disease was 33.6%, while it was higher in patients with cardiovascular disease. Approximately half of the participants with PhQ-9 scores indicating the presence of depression had diabetes, while one-third of hypertensive patients had PHQ-9 scores indicative of depression. The percentage of depression among participants taking diabetes, hypertension, and depression medications was 52.2%, 31.9%, and 39.3%, respectively. Consequently, medications can be considered a critical indicator that could affect biomarker levels. In this context, 45% of the participants did not take any medication, while 4.5% took three types of medication simultaneously and 17% used two types of medication.

**Figure 2:**
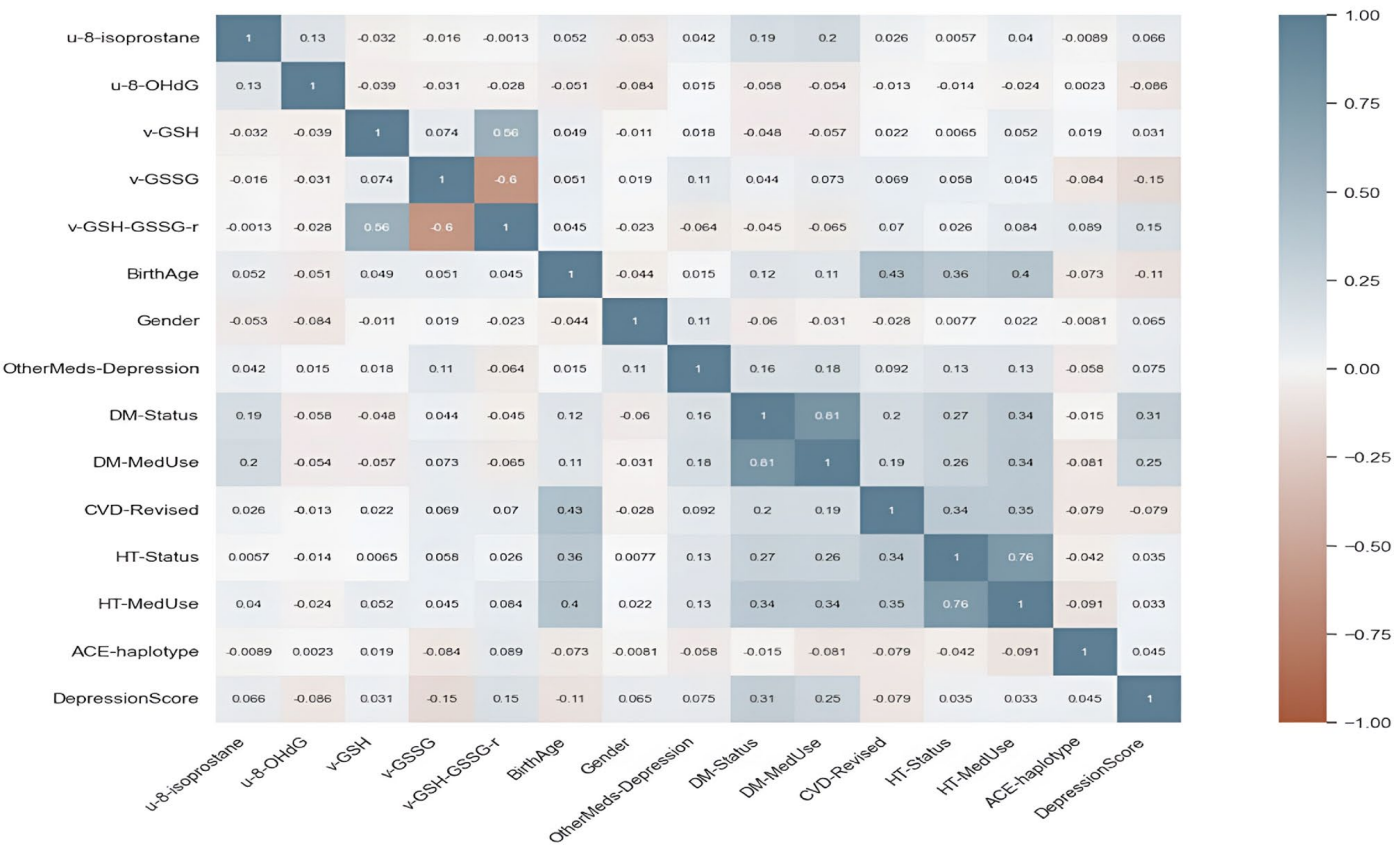
Pearson correlation matrix.

Figure 2 shows the Pearson correlation matrix among all features included in this study. Two variables are highly correlated if Pearson’s correlation coefficient is greater than 0.8, moderate if between 0.5 and 0.8, weak between 0.3 and 0.5, and no correlation if less than 0.3^29^. The correlation matrix indicated a slightly nonsignificant negative correlation between 8-isoprostane, 8-OHdG, GSH, and GSSG and depression. A very low negative correlation was observed between depression and age, while the presence of cardiovascular disease, diabetes, hypertension, and medication use showed a moderate positive correlation.

## Methods

**Figure 3:**
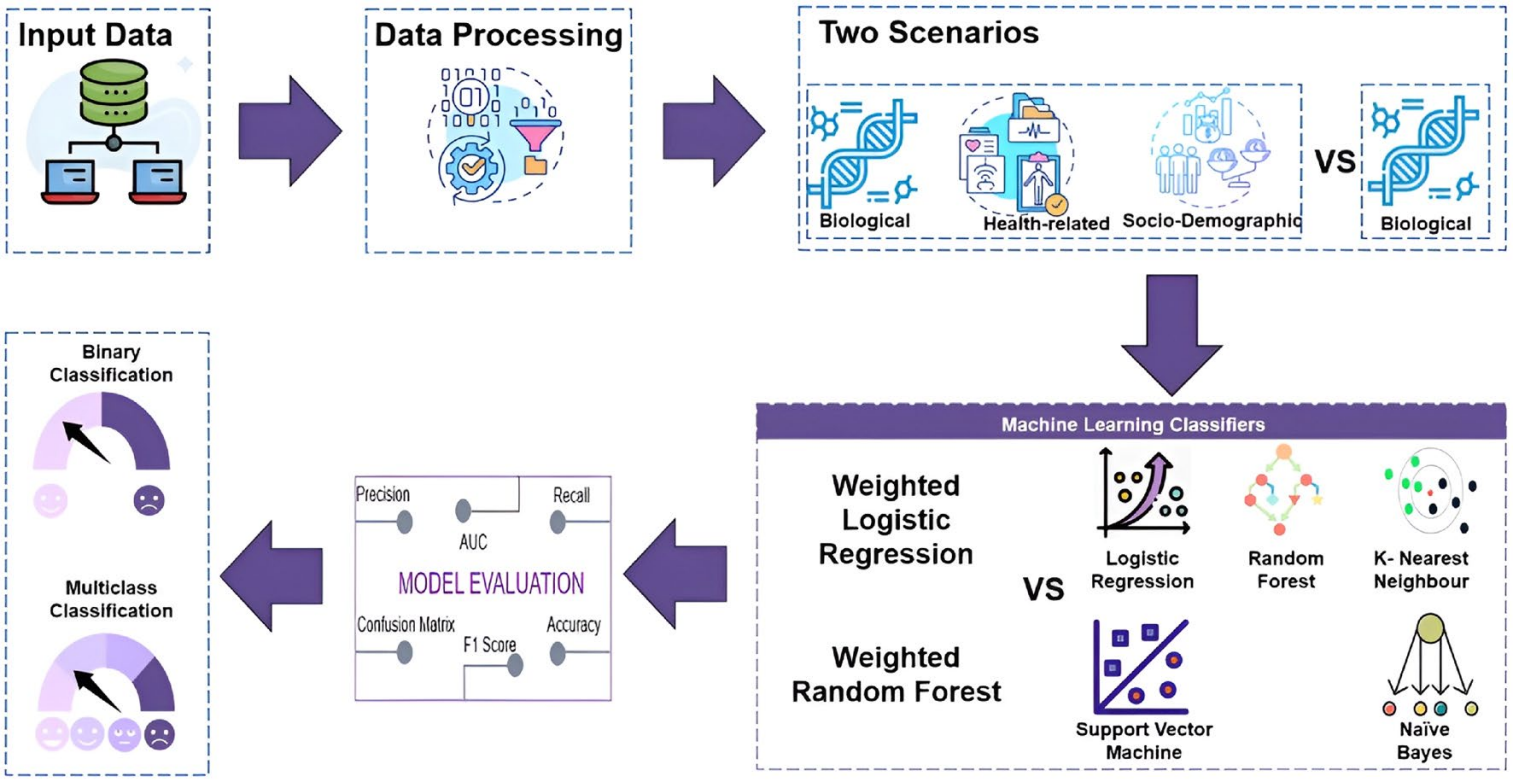
Framework for detecting depression severity.

Two classification models are proposed; binary classification to determine whether the patient has depression or not and multiclass classification to detect the level of depression. Different combinations of factors were tested to determine the optimal combination of factors that contribute to the occurrence of depression and their importance. Biomarkers of oxidative stress [8-isoprostane, 8-OHdG, GSH, GSSG in addition to the glutathione ratio (GSH-GSSG)] were included in the first model as the main features. In the second model, all biomarkers, sociodemographic, genetic and health-related characteristics were included. Data imputation was performed following Jelinek et al.^44^ where classes are defined by the Cartesian product of class values and incomplete information dismissal and data completion techniques are applied to reduce features and impute missing values. This method comprehensively handles missing values in clinical data sets by considering all possible combinations of class values, filtering out incomplete information, and employing data competition to fill in missing values, thereby creating a more complete and reliable data set for analysis. In addition, it has proven effective in feature reduction and missing value imputation in similar clinical settings, improving the detection accuracy of data mining algorithms. Figure 3 shows a pictorial representation of the entire methodology.

Python and Scikit-learn libraries were used to implement machine learning algorithms. Firstly, the data set has been split into training and testing data. This study has used 70% of the data set as training data and 30% for testing. To improve machine learning models, fivefold cross-validation with grid search over hyperparameter values in the training set (“Multimedia Appendix 1”). The optimal hyperparameters found for each model during the training phase were then used to evaluate the model’s performance on the testing set. The performance of machine learning models has been evaluated primarily using the AUC. Additional performance metrics such as recall, precision, F1 score, the area under the Precision-Recall Curve (AUC-PR) and confusion matrices were also reported. SMOTE, WLR and WRF have been implemented to improve detection performance given the class imbalance in the data set, achieving higher accuracy. To identify the relative importance of different factors, we determine the weight assigned to each factor, which reflects the contribution of each factor to the final detection. To evaluate the importance of features in our study, we used the permutation importance technique available in the scikit-learn library^40^. Permutation importance measures the influence of individual features on the model’s performance by randomly permuting the values of each feature and measuring the subsequent change in performance. This provides valuable information on the contribution of each feature to the overall detection of the severity of depression^45^. In this study, we considered the importance of the model feature with the highest accuracy.

### Data pre-processing

We extracted common features from the data sets and used a standardization procedure to ensure that the variables exhibited a mean of zero and a standard deviation of one. This standardization ensures a consistent scale across the data set, which is essential for machine learning models. By eliminating the influence of varying scales, it improves model robustness and reduces sensitivity to scale variability.

Subsequent to the standardization procedure, the data set was partitioned into a training set (70%) and a testing set (30%). The training set served as the basis for model development, while the testing set was used to evaluate the performance of the resulting trained model. To avoid overfitting of machine learning models, a fivefold cross-validation technique coupled with grid search across hyperparameter values was implemented in the training set (see “Multimedia Appendix 1”), in order to determine optimal configurations for each model.

### Machine learning models

In this study, we used Logistic Regression (LR), Random Forest (RF), K-Nearest Neighbour (K-NN), Support Vector Machine (SVM), Naïve Bayes (NB), and Artificial Neural Network (ANN). These models, known for their efficacy, have shown high performance in previous studies focusing on predicting and detecting the severity of depression, as reported in the existing literature.

#### Logistic regression (LR)

LR is used for classification problems where the goal is to determine whether a new sample fits in a particular class. It is useful for binary classification that can be generalized to multinominal outcomes^11,46^. It also has the ability to predict the probability of a specific class^47^. Mathematically, the logistic function is given by Eq. (1). There are variants of LR to help overcome the problem of overfitting, such as $L_1$ and $L_2$ regularization. $L_1$ regularization, also known as Lasso regularization, adds a penalty equal to the absolute value of the coefficients to the loss function. On the other hand, $L_2$ regularization, or Ridge regularization, adds a penalty equal to the square of the coefficients to the loss function. In this study, both variants of LR were utilized.

1 $$\begin{aligned} F(x) = \frac{1}{1 + e^{-(\beta _{0} + \beta _{1}x_{1} + \beta _{2}x_{2} + \beta _{3}x_{3} + \cdots )}} \end{aligned}$$

where $x$ is the input variable, $\beta _{0}$ is the intercept and $\beta _{1}, \beta _{2}, \beta _{3}, \ldots$ are the slopes of the logarithmic odds as a function of $x$.

#### Random forest (RF)

RF is a predictive modeling tool that builds decision trees and determines the average of the predictions of each decision tree^48^. Consequently, it combines simplicity and flexibility to increase predictive accuracy^49^. The RF algorithm has some stochastic behavior. The algorithm randomly selects samples from the original data set and creates a decision tree. It continuously repeats the creation of decision trees considering an independent subset of variables every time from the original data set, resulting in a wide variety of trees. This variety makes RF more effective than an individual decision tree model^50^.

Furthermore, randomness in the generation of decision trees increases the generalizability of RF so that the classifier is less likely to overfit^51^. Built-in cross-validation is one of the characteristics of RF that adds value to allow the classification of variables from the most effective to the least associated with the outcome variable. However, to obtain high classification accuracy from the model, it is important to increase the amount of data so that different classes can be distinguished well from each other^11^.

#### K-nearest-neighbor (K-NN)

KNN is a supervised machine learning algorithm that is widely used for both regression and classification. It is an effective algorithm when dealing with data sets with linear or nonlinear relationships. It assumes that similar data are close to each other. Consequently, KNN classifies new data points based on their proximity to the most similar instances in the data set^52^. Three parameters are used. N neighbors, which indicates the number of neighbors required for classification, the distance metric and the p-value^53^.

#### Support vector machine (SVM)

SVM is a machine learning algorithm for regression and classification. It has been widely used in the bioinformatics field for its effectiveness in handling nondimensional data and its robustness when dealing with outliers^54^. This algorithm uses a hyperplane to classify future predictions. The hyperplane can be represented as a line or a plane in multidimensional space to classify the data into the corresponding classes by investigating the maximum space margin between the support vectors^53^.

In the context of SVM, when working with a training data set containing (n) data points, denoted as $\{(x_1, y_1), \ldots , (x_n, y_n)\}$, where each $x_i$ is a sample in the n-dimensional input space associated with a binary output value $y_i \in \{1, 0\}$, for each $i = 1, 2, \ldots , n$, the SVM optimization problem can be mathematically expressed as follows (Eq. 2):

2 $$\begin{aligned} \text {Objective: } \text {Minimize } \frac{1}{2} \Vert \beta \Vert ^2 + C \sum _{i=1}^{n} \xi _i \end{aligned}$$

3 $$\begin{aligned} \text {Subject to:} \quad&y_i (\langle x_i, \beta \rangle + \beta _0) \ge 1 - \xi _i, \quad i = 1, 2, \ldots , n \end{aligned}$$

4 $$\begin{aligned}&\xi _i \ge 0, \quad i = 1, 2, \ldots , n \end{aligned}$$

Where $C$ serves as a constant that penalizes errors, and $\xi _i$ represents slack variables that indicate the extent of misclassification; if an instance is misclassified, then $\xi _i > 1$.

#### Naive Bayes (NB)

NB is a probabilistic supervised machine learning classification algorithm based on the Bayes theorem. It applies conditional probability between features given the values of class variables. This algorithm determines the probability of events based on the occurrence of previous events assuming independent features^53^.

#### Artificial neural network (ANN)

**Figure 4:**
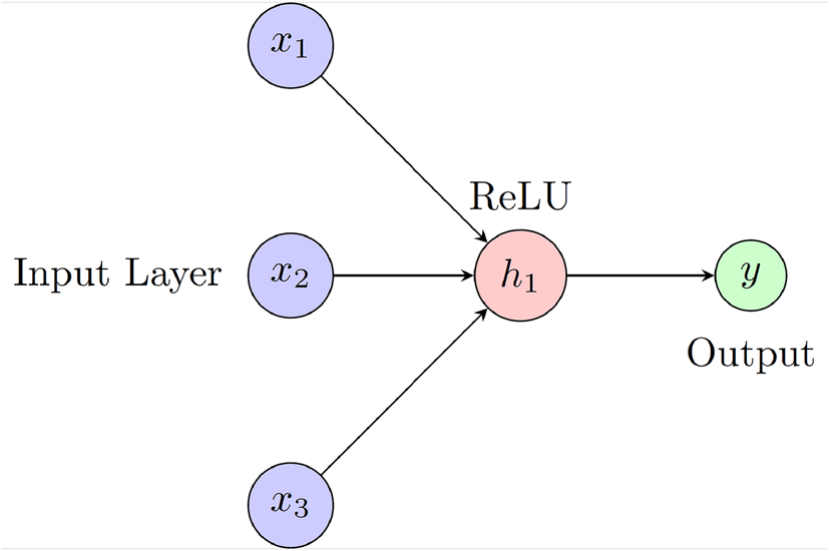
ANN architecture with three inputs, one output and RELU activation function.

ANN is a deep learning algorithm inspired by the structure and function of the human brain. It consists of interconnected layers, each layer consisting of neurons (as shown in Fig. 4). Each neuron incorporates an activation function^55^. ANN used three layers with a rectified linear unit activation function (RELU) with 350 epochs to train the model.

### Performance metrics

#### Accuracy

Accuracy is a performance metric that can be used to identify the percentage of correctly classified predictions. It can be expressed by Eq. (5).

5 $$\begin{aligned} \text {Accuracy} = \frac{\text {Number of Correct Predictions}}{\text {Total Number of Predictions}} \end{aligned}$$

#### Precision

Precision is used to identify the percentage of positive attempts that were correctly classified against the total number of positive predictions and can be expressed by Eq. (6).

6 $$\begin{aligned} \text {Precision} = \frac{\text {True Positives (TP)}}{\text {True Positives (TP) + False Positives (FP)}} \end{aligned}$$

#### Recall

Recall is used to calculate the ratio of positive predicted outcomes to the total predictions in a given class (Eq. 7)^48^.

7 $$\begin{aligned} \text {Precision} = \frac{\text {True Positives (TP)}}{\text {True Positives (TP) + False Positives (FN)}} \end{aligned}$$

#### F1-score

F1 score is a better performance metric as it considers both recall and precision, particularly for imbalanced data (data with nonuniform distribution of class labels). It can be determined by the harmonic mean of recall and precision^53^ as presented in Eq. (8).

8 $$\begin{aligned} \text {F1score} = \frac{\text {2(Recall + Precision)}}{\text {Recall + Precision}} \end{aligned}$$

#### Confusion matrix

The confusion matrix is a performance measurement tool that provides a breakdown of the predicted and actual outcomes of a classification model. It offers an in-depth understanding of the performance of any classification model, particularly in contexts where the implications of false positives and false negatives vary^53^.

#### Area under the ROC curve (AUC)

The Area Under the Curve of the Receiver Operating Characteristic (AUC-ROC) is a performance metric that quantifies the overall ability of a classification model to discriminate between classes. The ROC curve itself plots the True Positive Rate (TPR) against the False Positive Rate (FPR) across various classification thresholds, providing a visual representation of the model’s performance. The AUC value, which represents the area under this curve, serves as a measure of the accuracy of the model^56^. In the context of multi-class classification models, the (Macro-average) is employed as a performance metric. This approach calculates the average performance across all classes, treating each class with equal importance, which is particularly beneficial when dealing with imbalanced data sets.

#### Area under the precision–recall curve (AUC-PR)

The area under the precision-recall curve (AUC–PR) is an essential metric for assessing the performance of classifiers, especially in situations involving imbalanced data sets where positive class is rare. In contrast to the ROC curve, which plots the True Positive Rate (TPR) against the False Positive Rate (FPR), the Precision-Recall (PR) curve emphasizes precision and recall. This focus makes the PR curve more informative for imbalanced data scenarios. A higher AUC-PR value signifies superior performance, indicating the classifier’s ability to maintain both high precision and high recall across various thresholds^57^.

#### Statistical tests between different models

The Friedman test was used on the training data to evaluate the significant performance differences in accuracy among the various models and classifiers used in this study. The training data was split using a tenfold cross-validation method, and the resultant performance of the ten groups was analyzed using the Friedman test. The Friedman test^58^, a non-parametric statistical test, calculates a chi-square statistic and the corresponding p-values to determine statistical significance between the models. Subsequently, pairwise comparisons were made using the Conover post hoc test, implemented through the $scikit\_posthocs$ library, to assess significant differences between individual model pairs.

### Class imbalance

In the context of the development of prediction models within healthcare settings, the mitigation of class imbalance emerges as a crucial concern to ensure robust and impartial model performance. Class imbalance occurs when there is an uneven distribution of target classes in the data set, posing the risk that the model favors the majority class at the expense of overlooking significant minority classes. Accurate prediction or classification of depression is particularly important in clinical practice, influencing the determination of appropriate treatment strategies and optimal results.

### Class weight

To this end, another approach is proposed to improve the accuracy of the predictive models and avoid the effect of class imbalance. This approach is derived from the incorporation of class weight, which is based on penalizing the algorithm for incorrect prediction by placing a heavier penalty on misclassifying the minority class. Each class is assigned a weight, but minority classes are given larger weights (higher misclassification penalty)^59,60^as represented by Eq. (9) below:

9 $$\begin{aligned} W_j = \frac{n}{K \times n_j} \end{aligned}$$

where $W_j$ is the weight of class $j$, $n$ is the total number of observations, $K$ is the number of classes, and $n_j$ is the number of observations in class $j$.

### Synthetic minority over-sampling technique (SMOTE)

SMOTE is a widely adopted method for addressing class imbalance in classification tasks. SMOTE tackles this issue by generating synthetic examples for the minority class rather than simply replicating existing instances. In doing so, SMOTE effectively increases the sample size of the minority class and promotes a more balanced class distribution. This synthetic augmentation helps mitigate the overfitting associated with random oversampling and improves the classifier’s ability to generalize^61^.

## Results

### Binary classification

For binary classification, participants were grouped into two categories; PHQ-9 between 0 and 4 was considered to be without depression (class 1), and more than four on the PHQ-9 scale were classified as the presence of depression regardless of its severity (class 2).

**Figure 5:**
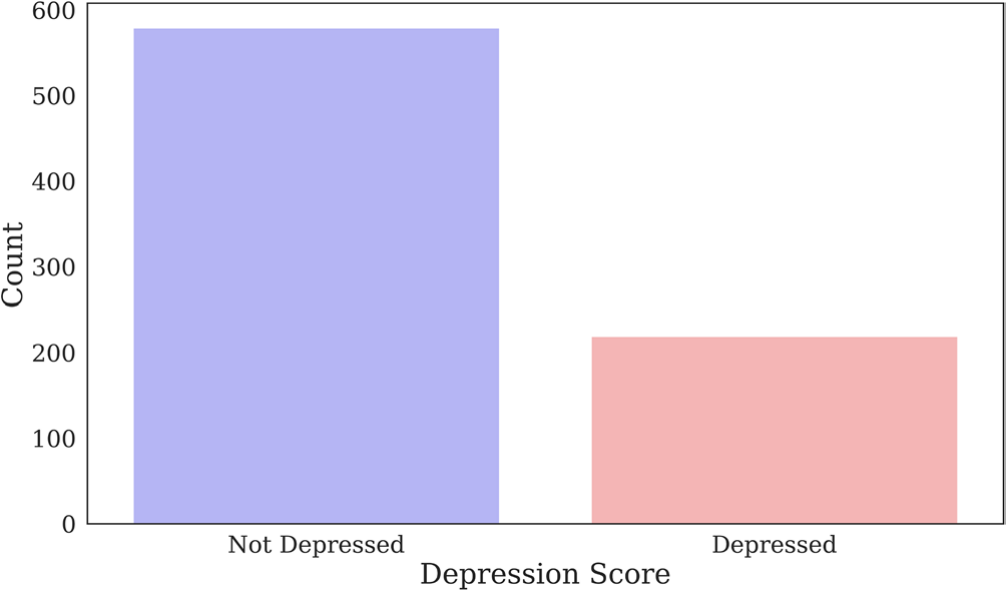
Distribution of depressed and not depressed participants.

The severity of depression, like other chronic diseases, is more often present in its mild form. Therefore, a critical part of data preparation was to check whether the two groups were matched in terms of participants. Figure 5 represents the distribution of depressed and non-depressed participants in the data set as a binary classification. As can be seen, 30.2% of the study population was classified as depressed, while 69.8% of the participants were not.

#### Binary classification using oxidative stress biomarkers

In this scenario, binary machine learning models were trained by including only oxidative stress markers. The performance metrics of the classifiers on the testing data (accuracy, precision, recall, F1 score, AUC, and AUC-PR are shown in Table 3. RF classifier had the best performance measures with an accuracy of 0.81, AUC of 0.81 and AUC-PR of 0.76, while the lowest classification performance was achieved with NB.

**Table 3:** Performance of the binary classifiers using five oxidative stress biomarkers as main features on the testing data.

Model	Accuracy	Precision	Recall	F1 Score	AUC	AUC–PR
LR	0.70	0.50	0.70	0.58	0.66	0.40
RF	0.81	0.80	0.80	0.80	0.81	0.76
KNN	0.78	0.77	0.78	0.77	0.81	0.70
SVM	0.78	0.82	0.78	0.74	0.72	0.56
NB	0.69	0.66	0.69	0.66	0.65	0.70
ANN	0.74	0.73	0.74	0.73	0.66	0.74

Using RF, the contribution of the variables of oxidative stress to the classification is shown in Table 4. The highest importance value was given to GSSG, followed by 8-OHdG and 8-isoprostane.

**Table 4:** Feature importance in RF for binary classification-oxidative stress.

Feature	Importance value (RF)
GSSG	0.213
8-OhdG	0.213
8-Isoprostane	0.211
GSH-GSSG-r	0.185
GSH	0.177

#### Binary classification using sociodemographic health related and oxidative stress biomarkers

The second model developed also included age, gender, chronic disease and medication use, and ACE genetics. Table 5 compares the performance metrics for the second model in the testing data set. The accuracy of all machine learning models was considered improved. RF outperforms other models with an accuracy of 0.83, an AUC of 0.84, and an AUC–PR of 0.78. Although the inclusion of sociodemographic and clinical factors in conjunction with oxidative stress has resulted in an incremental improvement in the binary classification, these features contributed to a much lower extent. This is highlighted by features importance, which ranks all oxidative stress biomarkers at the top, with ACE genetics in the third place in this model. Multimedia Appendix 2 presents the confusion matrices of all binary classification models (Table 6).

**Table 5:** Performance of the binary classifiers using socio-demographic, health-related, and oxidative stress biomarkers.

Model	Accuracy	Precision	Recall	F1 Score	AUC	AUC–PR
LR	0.79	0.77	0.78	0.76	0.76	0.59
RF	0.83	0.83	0.83	0.83	0.84	0.78
KNN	0.78	0.77	0.78	0.77	0.79	0.67
SVM	0.73	0.80	0.73	0.64	0.71	0.57
NB	0.72	0.72	0.72	0.72	0.74	0.57
ANN	0.77	0.78	0.77	0.77	0.74	0.78

**Table 6:** Features importance in RF for binary classification-all factors.

Feature	Importance value (RF)
GSH	0.147
8-Isoprostane	0.134
ACE haplotype	0.133
8-OHdG	0.122
GSSG	0.129
GSH-GSSG-r	0.111
BirthAge	0.061
DM-MedUse	0.034
DM-Status	0.028
HT-MedUse	0.023
HT-Status	0.021
OtherMeds-Depression	0.019
CVD	0.019
Gender	0.018

### Multiclass classification

**Figure 6:**
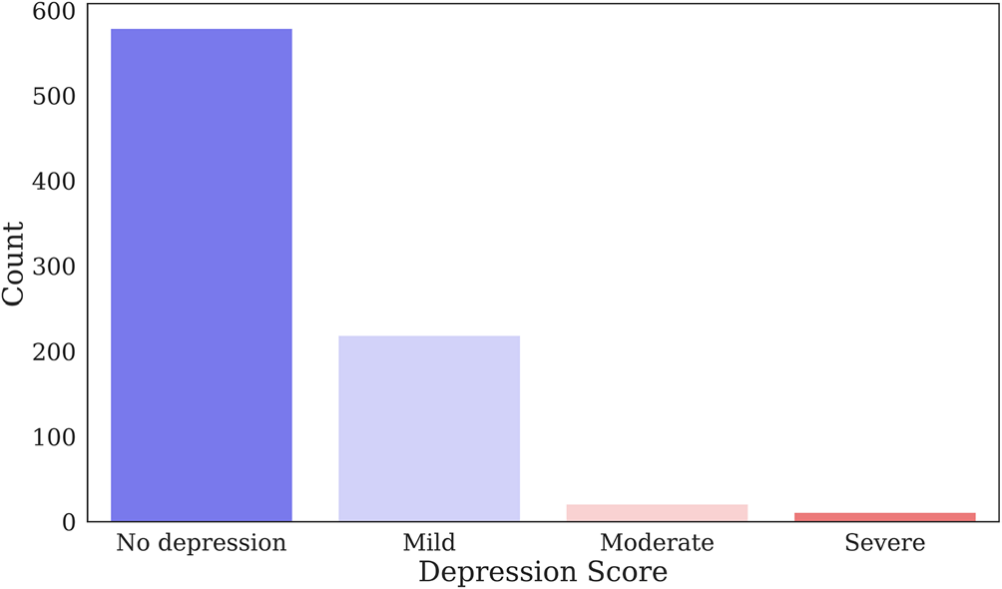
Distribution of multiclass depression levels among participants.

For treatment purposes, it is important to detect the level or severity of depression. Patients who were included in the analysis were classified into four groups: 0–4, no depression; 5–13, mild depression; 14–20, moderate depression; and 21 or greater, severe depression, according to the PHQ-9 criteria^43^. Figure 6 shows the distribution of participants among the four classes.

#### Multiclass classification using oxidative stress biomarkers

The five oxidative stress biomarkers were selected as the main features. Performance metrics were used to compare the results of the different machine learning algorithms. Table 7 compares the performance metrics of the machine learning algorithms used for multiclass classification in the testing data set. The highest AUC of 0.84 and AUC-PR of 0.88 were obtained with the RF classifier compared to approximately 0.58 for the ANN.

**Table 7:** Performance of the multiclass classifiers using five oxidative stress biomarkers as main features.

Model	Accuracy	Precision	Recall	F1 Score	AUC	AUC–PR
LR	0.70	0.71	0.70	0.58	0.61	0.73
RF	0.79	0.78	0.79	0.77	0.84	0.88
KNN	0.75	0.74	0.75	0.73	0.78	0.80
SVM	0.76	0.78	0.76	0.70	0.70	0.73
NB	0.67	0.65	0.67	0.66	0.71	0.66
ANN	0.70	0.70	0.70	0.70	0.58	0.59

Table 8 shows the ranking of the feature importance for RF. RF ranks 8-isoprostane as the highest, while the lowest was GSH similar to the binary model when only oxidative stress markers are included.

**Table 8:** Features importance—oxidative stress.

Feature	Importance value (RF)
8-Isoprostane	0.218
8-OHdG	0.217
GSSG	0.204
GSH-GSSG-r	0.194
GSH	0.167

#### Multiclass classification using socio-demographic—health related and oxidative stress biomarkers

The remaining sociodemographic and health-related factors were included in this model to investigate the performance of the multiclass models. Consequently, the analysis was repeated using the fourteen features and applying the same machine learning algorithms. Table 9 presents the performance measures in terms of accuracy, precision, recall, F1 score, AUC, and AUC-PR on the testing data set.

**Table 9:** Performance of the multiclass classifiers using sociodemographic, health-related, and oxidative stress biomarkers.

Model	Accuracy	Precision	Recall	F1 Score	AUC	AUC–PR
LR	0.73	0.73	0.74	0.69	0.73	0.80
RF	0.82	0.81	0.81	0.79	0.88	0.90
KNN	0.76	0.75	0.76	0.74	0.77	0.83
SVM	0. 67	0.75	0.67	0.55	0.65	0.75
NB	0.65	0.67	0.65	0.65	0.66	0.60
ANN	0.73	0.72	0.73	0.72	0.63	0.69

All classification models showed improvement compared to the model with only oxidative markers except NB, with RF having an AUC of 0.88 compared to 0.84. The role and importance of oxidative stress biomarkers remained significant for the model with the highest importance values. Table 10 shows the feature importance values for the RF classifier. ACE genetics is the most important feature in the classification, while gender was ranked the least. The confusion matrices of the different algorithms are shown in Multimedia Appendix 2.

**Table 10:** Feature importance—all factors.

Feature	Importance value (RF)
ACE haplotype	0.149
GSH	0.137
8-Isoprostane	0.136
GSSG	0.130
8-OHdG	0.127
GSH-GSSG-r	0.120
BirthAge	0.045
DM-Status	0.032
HT-Status	0.022
OtherMeds-Depression	0.022
DM-MedUse	0.021
HT/CVD-MedUse	0.021
CVD-Status	0.020
Gender	0.019

### Statistical comparison among machine learning models

The Friedman test was applied to evaluate the significance of performance differences between models. Given the superior performance of RM among all models, we subsequently conducted a pairwise comparison using the Conover post hoc test, specifically between RF and all other algorithms. The Chi-square for the Friedman test and the P-values of all models are presented in Table 11. The resulting values of the Chi-square and the P-value derived from the Friedman test imply significant differences between the algorithms. Furthermore, the results of the post hoc Conover analysis between RF and all other alternatives demonstrate the marked improvement of RF over others at $\alpha = 0.05$. However, it should be noted that the statistical difference was not significant with SVM when only oxidative stress biomarkers were used for binary and multiclass classifications.

**Table 11:** Statistical comparison test results between different algorithms.

	Binary classification		Multiclass classification	
	Oxidative stress	All features	Oxidative stress	All features
Friedman test among all models				
Chi-square	40.85	30.93	47.19	36.32
P-value	$1.00 \times 10^{-7}$	$9.65 \times 10^{-6}$	$5.2 \times 10^{-9}$	$8.2 \times 10^{-7}$
Conover post-hoc test				
P-value RF-LR	$7.96 \times 10^{-8}$	$0.96 \times 10^{-5}$	$1.13 \times 10^{-3}$	$0.10 \times 10^{-4}$
P-value RF-SVM	$1.16 \times 10^{-1}$	$0.40 \times 10^{-5}$	$1.55 \times 10^{-1}$	$0.90 \times 10^{-4}$
P-value RF-KNN	$2.83 \times 10^{-2}$	$0.38 \times 10^{-3}$	$6.62 \times 10^{-7}$	$3.25 \times 10^{-2}$
P-value RF-NB	$2.44 \times 10^{-11}$	$8.45 \times 10^{-7}$	$4.56 \times 10^{-21}$	$1.87 \times 10^{-12}$
P-value RF-ANN	$8.98 \times 10^{-15}$	$3.54 \times 10^{-10}$	$1.90 \times 10^{-25}$	$2.71 \times 10^{-9}$

### Weighted class logistic regression and random forest

#### Binary classification

**Figure 7:**
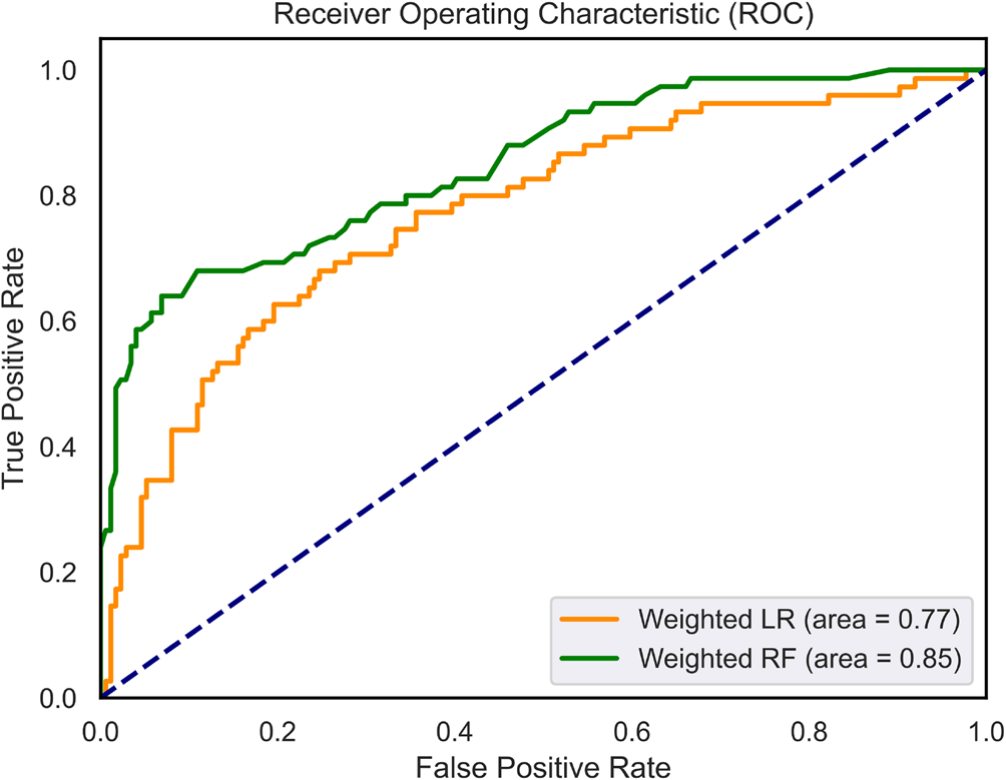
ROC curves of the weighted classifiers (WLR and WRF) for the binary models.

**Figure 8:**
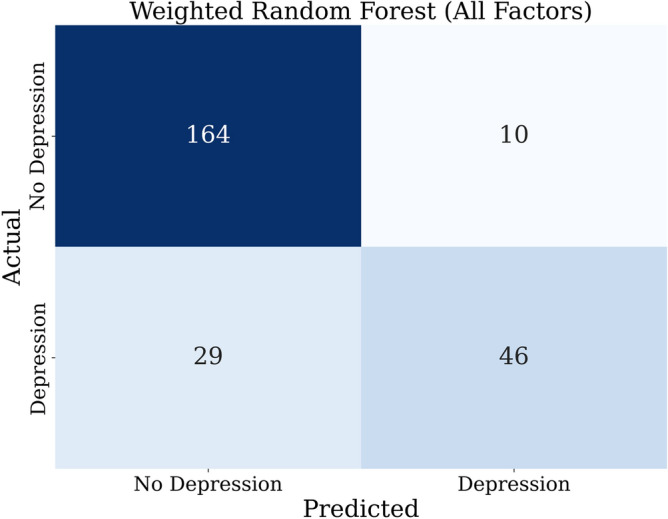
Confusion matrix of the weighted binary RF model with all features.

The weights for the detection of the binary class that includes all 14 characteristics were determined based on the Eq. (9) as 0.72 for class 1 and 1.65 for class 2. The application of WLR and WRF using all features has resulted in AUC values of 0.77 and 0.85, respectively, which are close to those without weighing. This might be attributed to the effectiveness of the weighted-class technique in tackling imbalanced data present in our multiclass distribution. In addition, a slight improvement in the quality of the detection in terms of smaller percentages of false positives has been achieved. Figure 7 compares the ROC curves of the binary classifiers for WLR and WRF using all features, indicating the outperforming performance of WRF compared to WLR. Figure 8 shows the resultant confusion matrix of the WRF of the binary classification model using all features.

Table 12, presents the important features of the WRF. In this model, oxidative stress biomarkers and ACE are the most valuable features, while gender, age, medication use, and comorbidities played a minor role in the classification of MDD and disease progression according to PHQ-9.

**Table 12:** Feature importance in RF for binary classification—all factors—WRF.

Feature	Importance value (RF)
GSH	0.154
GSSG	0.138
ACE haplotype	0.135
8-Isoprostane	0.129
8-OHdG	0.119
BirthAge	0.065
GSH-GSSG-r	0.113
DM-MedUse	0.027
HT-Status	0.0197
DM-Status	0.025
Gender	0.016
HT-MedUse	0.019
OtherMeds-Depression	0.018
CVD	0.016

#### Multiclass classification

**Figure 9:**
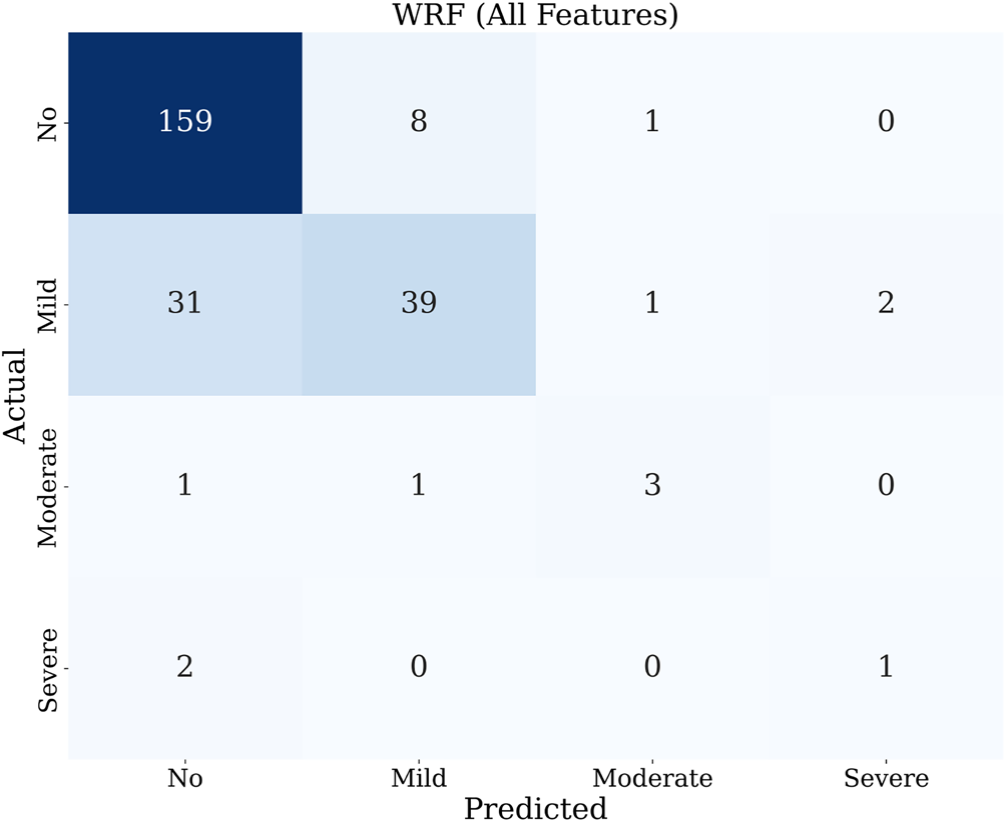
Confusion matrix of weighted RF with all features.

For this model, we measure the performance of the weighted algorithms in detecting the multi-class depression levels. Table 13 presents the measured accuracy, precision, recall, F1 score, AUC, and AUC–PR of applying the WLR, WRF, and RF with SMOTE algorithms to the testing data set. WRF has the best performance with AUC values of 0.85 and 0.91, considering only oxidative stress and all features, respectively. Both data mining models perform better than when no weighting is applied. Figure 9 shows the confusion matrix of the WRF algorithm utilizing all features. The rest of the confusion matrices are shown in Multimedia Appendix 2.

**Table 13:** Performance of weighted multiclass classifiers.

	Oxidative stress biomarkers			All features		
	WLR	WRF	RF with SMOTE	WLR	WRF	RF with SMOTE
Accuracy	0.70	0.79	0.69	0.70	0.82	0.76
Precision	0.66	0.78	0.74	0.72	0.81	0.77
Recall	0.70	0.79	0.69	0.70	0.82	0.76
F1 Score	0.65	0.78	0.70	0.70	0.80	0.76
AUC	0.65	0.85	0.86	0.73	0.91	0.80
AUC-PR	0.67	0.85	0.78	0.77	0.90	0.85

**Figure 10:**
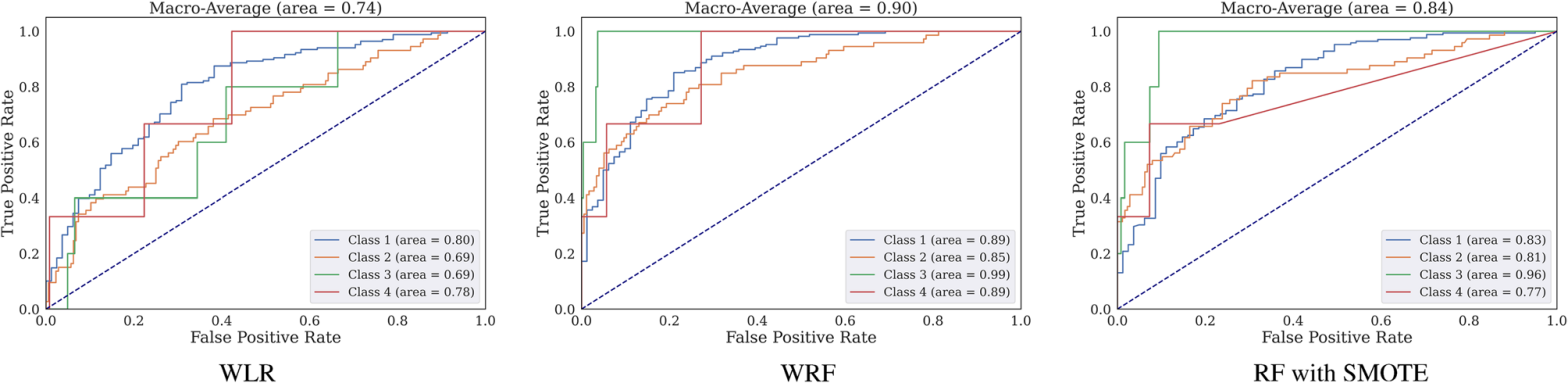
ROC curves of the weighted classifiers (WLR and WRF) and RF with SMOTE for the multi-class models and the (Macro-Average) AUC values.

Table 14 presents the important features used for the WRF. In this model, oxidative stress biomarkers, GSH in particular, are the most valuable features, while the presence of diabetes was the least. Figure 10 shows the ROC curves of the weighted multi-class classifiers (WRF and WLR) and RF with SMOTE using all 14 features, indicating a better performance of WRF than that of WLR and RF-SMOTE.

**Table 14:** Feature importance in RF for multiclass classification—all factors—WRF.

Feature	Importance value (RF)
v-GSH	0.132
v-GSSG	0.118
u-8-Isoprostane	0.155
u-8-OHdG	0.109
ACE haplotype	0.140
v-GSH-GSSG-r	0.105
DM-MedUse	0.032
BirthAge	0.051
HT-Status	0.024
HT-MedUse	0.032
CVD	0.012
Gender	0.024
OtherMeds-Depression	0.018
DM-Status	0.015

### Statistical comparison among machine learning weighted models

We applied the Friedman test for the weighted class models to assess the significant difference in the algorithm’s performance. Table 15 presents the resulting values obtained from the statistical tests. First, we compare the results of the WLR, WRF, and RF with SMOTE when using only oxidative biomarkers as the main features. Subsequently, a pairwise comparison was conducted using the Conover post-hoc test between the WRF and all different algorithms since it has the best performance. The same comparison was applied to the WLR, WRF, and RF with SMOTE when utilizing all features on the model. WRF showed significant improvement to different algorithms with a $\alpha = 0.05$ for both data mining models while RF with SMOTE with a $\alpha = 0.1$ when using all features.

In addition, we applied a statistical comparison to assess the significance of differences among various feature sets used in the data set. The WRF classifier was used in three scenarios: using only oxidative stress biomarkers, using only social and demographic features, and using all features in the data set. When comparing the WRF with all features with the other models, including all features resulted in a significant improvement compared to other models. Furthermore, the comparison of the WRF with all features to the models using only oxidative stress biomarkers and only social and demographic features revealed that using only oxidative stress biomarkers has resulted in significant improvement in performance compared to the use of only social and demographic features.

**Table 15:** Statistical comparison between the results for weighted models.

	WLR, WRF, and RF with SMOTE		WRF across various feature sets	
	Oxidative stress	All features		
Friedman test among all models				
Chi-square	13.63	13.40		17.21
P-value	$0.11 \times 10^{-2}$	$0.12 \times 10^{-2}$		$0.18 \times 10^{-3}$
Conover post-hoc test				
P-value (WRF-WLR)	$0.8 \times 10^{-5}$	$0.14 \times 10^{-4}$	P-value (All features Vs. oxidative stress)	$3.68 \times 10^{-4}$
P-value (WRF-RF-SMOTE)	$0.17 \times 10^{-2}$	$0.18 \times 10^{-1}$	P-value (All features Vs. social and clinical)	$4.25 \times 10^{-3}$
			P-value (oxidative stress vs. social and clinical)	$8.83 \times 10^{-6}$

## Discussion

The PHQ-9 score has previously been validated for the classification of depression and disease progression using machine learning tools^62^. A meta-analysis investigating the relationship between oxidative stress and depression has indicated a statistically significant effect of oxidative stress on depression despite the substantial heterogeneity of the findings due to differences in measures of depression and oxidative stress biomarkers^25^. The current study develops machine learning-based models to detect the existence of depression and its severity using oxidative stress biomarkers together with social and clinical characteristics. The study includes glutathione, 8-isoprostane, and 8-hydroxy-2’-deoxyguanosine (8-OHdG), which are among the biomarkers most used for oxidative stress^25^.

RF has consistently been shown to outperform all other models in various scenarios and metrics tested in our study. This result is consistent with previous studies in clinical settings^21,31^. Although ANN is a more sophisticated algorithm, it requires large data sets to train the model, which could be the reason why it did not perform as well as RF in this clinical sample. Another approach was implemented based on assigning a class weight to address imbalanced data. A significant improvement was achieved by applying WRF with an AUC value of 0.91 for multiclass classification. In fact, the weighted class method has resulted in almost the same accuracy and AUC for binary classification. This might be attributed to its effectiveness in dealing with imbalanced data.

The analysis of the feature importance validates the role of oxidative stress biomarkers in depression detection performance. Oxidative stress biomarkers were ranked first in terms of their importance in various scenarios, underscoring their role, particularly GSH, in contributing substantially to the presence of depression^11,12^. The ACE gene haplotype has also been highlighted for its influence on the efficacy of treatment with selective serotonin reuptake inhibitors and (SSRI)^50^ and its role in depression.

Interestingly, the statistical analysis showed a significant difference between the models, with a better improvement in the RF performance. Numerous previous studies have applied machine learning to predict depression based on social and clinical characteristics. However, the inclusion of all features demonstrated the best performance, highlighting the significant advantage of using biological, social, and clinical features over using partial feature sets in the data set.

The limitation of this study was the disproportionately low number of participants with severe depression compared to other classes. This imbalance limits our ability to practically validate our results and compare them to those obtained using the weighting-class method. Therefore, future research can expand upon this study by specifically targeting participants with severe depression to better understand and validate the detection models tailored to this specific class. Further research needs to incorporate deep learning and explainable artificial intelligence (XAI) techniques to refine and advance detection performance progressively.

## Conclusion

In this study, we develop a novel approach that integrates clinical, sociodemographic, and oxidative stress markers. Our statistical analysis shows the significant role of oxidative stress biomarkers in the detection of depression. The results revealed that this integration of oxidative stress biomarkers with other features contributes to an improved detection of the severity level of depression. The analysis of feature analysis ranks oxidative stress biomarkers the first among other features, underscoring the role that they could serve as a reliable marker for depression, which aligns with the literature that points to the potential benefits of antidepressants with anti-inflammatory properties. The use of weighted models such as WRF and WLR as well as SMOTE shows the usefulness of these methods in the presence of imbalanced data, which is common in medical research. WRF technique has shown better performance compared to SMOTE.

Our study introduced a detection model that integrates sociodemographic variables with a comprehensive database of biomarkers of oxidative stress. This makes a transition from previous research that the current study uses biological factors to detect depression, leading to an enhanced and more robust detection model. This synthesis not only deepens our understanding of the multiple factors that influence depression and disease progression, but also establishes the basis for future research aimed at improving the integration of machine learning into understanding and detecting depression and its severity levels.

### Supplementary Information

Supplementary Information.

## Data Availability

The data set containing the 830 entries with the features used for this study is available upon reasonable request from the corresponding author.

## References

[1] Hidaka BH (2012). Depression as a disease of modernity: Explanations for increasing prevalence. J. Affect. Disord..

[2] Institute of Health Metrics and Evaluation. *Global Health Data Exchange (ghdx)* (2022).

[3] World Health Organization. *Depressive Disorder (Depression)* (2022).

[4] Ashraf, A. *et al.**A Summarization of the Visual Depression Databases for Depression Detection*. 1–6. 10.1109/ICWT50448.2020.9243625 (2020).

[5] Saha B, Nguyen T, Phung D, Venkatesh S (2016). A framework for classifying online mental health-related communities with an interest in depression. IEEE J. Biomed. Health Inform..

[6] Kang, M., Oh, S., Oh, K., Kang, S. & Lee, Y. The deep learning method for predict Beck’s depression inventory score using EEG. In *2021 International Conference on Information and Communication Technology Convergence (ICTC)*. 490–493. 10.1109/ICTC52510.2021.9620922 (2021).

[7] Black, C. N., Bot, M., Scheffer, P. G., Cuijpers, P. & Penninx, B. W. Is depression associated with increased oxidative stress? A systematic review and meta-analysis. *Psychoneuroendocrinology* **51**, 164–175 10.1016/j.psyneuen.2014.09.025 (2015) (this issue includes a special section on biomarkers in the military—new findings from prospective studies). 10.1016/j.psyneuen.2014.09.025 25462890

[8] Wium-Andersen IK, Osler M, Jørgensen MB, Rungby J, Wium-Andersen MK (2022). Diabetes, antidiabetic medications and risk of depression—A population-based cohort and nested case–control study. Psychoneuroendocrinology.

[9] Maes, M., Galecki, P., Chang, Y. S. & Berk, M. A review on the oxidative and nitrosative stress (o &ns) pathways in major depression and their possible contribution to the (neuro)degenerative processes in that illness. *Prog. Neuro-Psychopharmacol. Biol. Psychiatry* **35**, 676–692 10.1016/j.pnpbp.2010.05.004 (2011) (the neuro-inflammatory and neuroprogressive pathways in depression). 10.1016/j.pnpbp.2010.05.004 20471444

[10] Bhatt S, Nagappa AN, Patil CR (2020). Role of oxidative stress in depression. Drug Discov. Today.

[11] Pouvreau C, Dayre A, Butkowski EG, de Jong B, Jelinek HF (2018). Inflammation and oxidative stress markers in diabetes and hypertension. J. Inflamm. Res..

[12] Hassan W (2016). Association of oxidative stress with psychiatric disorders. Curr. Pharmaceut. Des..

[13] Wicke F (2022). The association of depression and all-cause mortality: Explanatory factors and the influence of gender. J. Affect. Disord..

[14] Tautan A-M, Ionescu B, Santarnecchi E (2021). Artificial intelligence in neurodegenerative diseases: A review of available tools with a focus on machine learning techniques. Artif. Intell. Med..

[15] Triantafyllidis A, Tsanas A (2019). Applications of machine learning in real-life digital health interventions: Review of the literature. J. Med. Internet Res..

[16] Pienaar MA, Sempa JB, Luwes N, Solomon LJ (2022). An artificial neural network model for pediatric mortality prediction in two tertiary pediatric intensive care units in South Africa. A development study. Front. Pediatr..

[17] Nemesure M, Heinz M, Huang R, Jacobson N (2021). Predictive modeling of depression and anxiety using electronic health records and a novel machine learning approach with artificial intelligence. Sci. Rep..

[18] Kaushik P, Yang H, Roy P, Vugt M (2023). Comparing resting state and task-based EEG using machine learning to predict vulnerability to depression in a non-clinical population. Sci. Rep..

[19] Cvetkovic J (2017). Breast cancer patients’ depression prediction by machine learning approach. Cancer Invest..

[20] Sau A, Bhakta I (2017). Predicting anxiety and depression in elderly patients using machine learning technology. Healthc. Technol. Lett..

[21] Priya, A., Garg, S. & Tigga, N. P. Predicting anxiety, depression and stress in modern life using machine learning algorithms. *Proc. Comput. Sci.* **167**, 1258–1267 10.1016/j.procs.2020.03.442 (2020) (international conference on computational intelligence and data science).

[22] Chao H-Y, Hsu C-H, Wang S-T, Yu C-Y, Chen H-M (2021). Mediating effect of social support on the relationship between illness concealment and depression symptoms in patients with pulmonary arterial hypertension. Heart Lung.

[23] Nguyen H, Oh H, Kim M-S (2021). The association between curry-rice consumption and hypertension, type 2 diabetes, and depression: The findings from Knhanes 2012–2016. Diabetes Metab. Syndr. Clin. Res. Rev..

[24] Zheng H, Jia C (2022). Gender differences in the association of depression trajectories with executive and memory functions: Evidence from the longitudinal study of the survey of health, ageing and retirement in Europe (2004–2017). J. Psychiatr. Res..

[25] Terauchi M (2016). Depressive symptoms are associated with oxidative stress in middle-aged women: A cross-sectional study. BioPsychoSoc. Med..

[26] Tuura R (2023). Prefrontal glutathione levels in major depressive disorder are linked to a lack of positive affect. Brain Sci..

[27] Maes M (2009). Increased 8-hydroxy-deoxyguanosine, a marker of oxidative damage to DNA, in major depression and myalgic encephalomyelitis/chronic fatigue syndrome. Neuro Endocrinol. Lett..

[28] Forlenza MJ, Miller GE (2006). Increased serum levels of 8-hydroxy-2’-deoxyguanosine in clinical depression. Psychosom. Med..

[29] Chen, H. & Chang, X. Photovoltaic power prediction of LSTM model based on Pearson feature selection. *Energy Rep.* **7**, 1047–1054. 10.1016/j.egyr.2021.09.167 (2021) (2021 international conference on energy engineering and power systems).

[30] Richter T, Fishbain B, Markus A, Richter-Levin G, Okon-Singer H (2020). Using machine learning-based analysis for behavioral differentiation between anxiety and depression. Sci. Rep..

[31] Na K-S, Cho S-E, Geem ZW, Kim Y-K (2020). Predicting future onset of depression among community dwelling adults in the Republic of Korea using a machine learning algorithm. Neurosci. Lett..

[32] Hooker SA (2022). Depression and cardiovascular risk in primary care patients. J. Psychosom. Res..

[33] Li H, Liu X, Zheng Q, Zeng S, Luo X (2022). Gender differences and determinants of late-life depression in China: A cross-sectional study based on Charls. J. Affect. Disord..

[34] Kodydková J (2009). Antioxidative enzymes and increased oxidative stress in depressive women. Clin. Biochem..

[35] Cabello M (2020). The relationship between all-cause mortality and depression in different gender and age groups of the Spanish population. J. Affect. Disord..

[36] Shen J (2024). A diagnostic model based on bioinformatics and machine learning to differentiate bipolar disorder from schizophrenia and major depressive disorder. Schizophrenia.

[37] Zulfiker M, Ety N, Biswas AA, Nazneen T, Uddin MS (2021). An in-depth analysis of machine learning approaches to predict depression. Curr. Res. Behav. Sci..

[38] Nandanwar, H. & Nallamolu, S. Depression prediction on twitter using machine learning algorithms. In *2021 2nd Global Conference for Advancement in Technology (GCAT)*. 1–7. 10.1109/GCAT52182.2021.9587695 (2021).

[39] Chawla N, Bowyer K, Hall L, Kegelmeyer W (2002). Smote: Synthetic minority over-sampling technique. J. Artif. Intell. Res. (JAIR).

[40] Pedregosa F (2012). Scikit-learn: Machine learning in Python. J. Mach. Learn. Res..

[41] Maalouf M, Trafalis TB (2011). Robust weighted kernel logistic regression in imbalanced and rare events data. Comput. Stat. Data Anal..

[42] Jelinek H, Wilding C, Tinely P (2006). An innovative multi-disciplinary diabetes complications screening program in a rural community: A description and preliminary results of the screening. Aust. J. Prim. Health.

[43] Ye Y-X (2022). Associations between depression, oxidative stress, and semen quality among 1,000 healthy men screened as potential sperm donors. Fertil. Steril..

[44] Jelinek H, Yatsko A, Stranieri A, Venkatraman S (2014). Novel data mining techniques for incomplete clinical data in diabetes management. Br. J. Appl. Sci. Technol..

[45] Altmann A, Tolosi L, Sander O, Lengauer T (2010). Permutation importance: A corrected feature importance measure. Bioinformatics (Oxford, England).

[46] Maalouf M (2011). Logistic regression in data analysis: An overview. Int. J. Data Anal. Tech. Strateg..

[47] Chao-Ying Joanne Peng KLL, Ingersoll GM (2002). An introduction to logistic regression analysis and reporting. J. Educ. Res..

[48] Subasi, A. *Practical Machine Learning for Data Analysis Using Python* (2020).

[49] Zolbanin HM, Delen D, Hassan Zadeh A (2015). Predicting overall survivability in comorbidity of cancers: A data mining approach. Decis. Supp. Syst..

[50] Firouzabadi N, Farshadfar P, Haghnegahdar M, Alavi-Shoushtari A, Ghanbarinezhad V (2021). Impact of ace 2 genetic variant on antidepressant efficacy of SSRIS. Acta Neuropsychiatr..

[51] Hastie, T., Tibshirani, R. & Friedman, J. *The Elements of Statistical Learning: Data Mining, Inference, and Prediction. * 2nd Ed. (Springer Series in Statistics, 2009).

[52] Ali N, Neagu D, Trundle P (2019). Evaluation of k-nearest neighbour classifier performance for heterogeneous data sets. SN Appl. Sci..

[53] Churcher A (2021). An experimental analysis of attack classification using machine learning in IoT networks. Sensors.

[54] Pavlidis P, Wapinski I, Noble WS (2004). Support vector machine classification on the web. Bioinformatics.

[55] Goodfellow, I., Bengio, Y. & Courville, A. *Deep Learning*. http://www.deeplearningbook.org (MIT Press, 2016).

[56] Yuan Y, Su W, Zhu M (2015). Threshold-free measures for assessing the performance of medical screening tests. Front. Public Health.

[57] Keilwagen J, Grosse I, Grau J (2014). Area under precision-recall curves for weighted and unweighted data. PLOS ONE.

[58] Pereira D, Afonso A, Medeiros F (2015). Overview of Friedman’s test and post-hoc analysis. Commun. Stat.-Simul. Comput..

[59] Zhang, C., Li, Y., Yu, Z. & Tian, F. *A Weighted Random Forest Approach to Improve Predictive Performance for Power System Transient Stability Assessment.* 1259–1263. 10.1109/APPEEC.2016.7779695 (2016).

[60] Maalouf M, Siddiqi M (2014). Weighted logistic regression for large-scale imbalanced and rare events data. Knowl.-Based Syst..

[61] Chawla NV, Bowyer KW, Hall LO, Kegelmeyer WP (2002). Smote: Synthetic minority over-sampling technique. J. Artif. Int. Res..

[62] Lopresti AL, Maker GL, Hood SD, Drummond PD (2014). A review of peripheral biomarkers in major depression: The potential of inflammatory and oxidative stress biomarkers. Prog. Neuro-Psychopharmacol. Biol. Psychiatry.

