# Supplementary material for: Detecting depression severity using weighted random forest and oxidative stress biomarkers
Source: Sci Rep. 2024 Jul 15;14:16328. doi: 10.1038/s41598-024-67251-y (PMC11250802; doi:10.1038/s41598-024-67251-y)
Supplement: Supplementary file 1 — Supplementary Information. [file 41598_2024_67251_MOESM1_ESM.pdf]

Multimedia Appendix 1. Hyperparameter tuning.

The hyperparameters of the ML classifiers obtained through the grid search cross-validation were as follows:

Table 1: Hyperparameter value range and optimal values for all ML methods.

Model	Hyperparameters	Best combination	
		All factors	Oxidative stress factors only
Binary Classification			
LR	C_ values: [0.001, 0.01, 0.1, 1, 10, 100]<br>Penalty: [l1, l2]<br>Solvers: liblinear, lbfgs, newton-cg, sag, saga]	C_value: 1<br>Penalty: l2<br>Solver: liblinear	C_value: 0.001<br>Penalty: l2<br>Solver: liblinear
RF	n_estimators: [50, 100, 150, 200, 300]<br>max_depth: [None, 5, 10, 15, 20, 25, 30]<br>min_samples_split: [2, 5, 10]<br>min_samples_leaf': [1, 2, 4]<br>bootstrap: [True, False]<br>max_features: [auto, sqrt, log2]<br>max_leaf_nodes: [None, 5, 10, 20, 50, 100]	n_estimators: 50<br>max_depth: 15<br>min_samples_split: 2<br>min_samples_leaf: 1<br>bootstrap: False<br>max_leaf_nodes: None	n_estimators: 50<br>max_depth: None<br>min_samples_split: 5<br>min_samples_leaf: 1<br>bootstrap: False<br>max_leaf_nodes: None
KNN	n_neighbors : [3, 5,7,9,11,13,15]<br>weights: [uniform, distance]<br>metric: [minkowski, euclidean, manhattan]	n_neighbors: 11<br>weights: distance<br>Metric: manhattan	n_neighbors: 15<br>weights: distance<br>Metric: manhattan
SVM	C: [0.1, 1, 10, 100]<br>gamma: [1, 0.1, 0.01, 0.001]<br>kernel: [linear, rbf]	C: 1<br>gamma: 0.1<br>kernel: rbf	C: 1<br>gamma: 1<br>kernel:rbf
NB	Naive Bayes has almost no hyperparameters to tune, so it usually generalizes well		
WLR	solvers = ['newton-cg', 'liblinear']<br>penalty = ['l1', 'l2']<br>c_values = [100, 10, 1.0, 0.1, 0.01]	solver: newton-cg<br>penalty: l2<br>C: 1	solver: liblinear<br>penalty: l2<br>C: 0.1
WRF	n_estimators: [50, 100, 150, 200, 300]<br>max_depth: [None, 5, 10, 15, 20, 25, 30]<br>min_samples_split: [2, 5, 10]<br>min_samples_leaf': [1, 2, 4]<br>bootstrap: [True, False]<br>max_features: [auto, sqrt, log2]<br>max_leaf_nodes: [None, 5, 10, 20, 50, 100]	n_estimators: 50<br>max_depth: None<br>min_samples_split: 2<br>min_samples_leaf: 1<br>bootstrap: True<br>max_leaf_nodes: None	n_estimators: 100<br>max_depth: 25<br>min_samples_split: 2<br>min_samples_leaf: 1<br>bootstrap: False<br>max_leaf_nodes: None
Multi-class Classification			

LR	solvers = ['newton-cg', 'lbfgs', 'liblinear']<br>penalty = ['l1', 'l2']<br>c_values = [100, 10, 1.0, 0.1, 0.01]	Solver: liblinear<br>Penalty: l2<br>C: 10	Solver: liblinear<br>Penalty: l2<br>C: 0.001
RF	n_estimators: [50, 100, 150, 200, 300]<br>max_depth: [None, 5, 10, 15, 20, 25, 30]<br>min_samples_split: [2, 5, 10]<br>min_samples_leaf': [1, 2, 4]<br>bootstrap: [True, False]<br>max_features: [auto, sqrt, log2]<br>max_leaf_nodes: [None, 5, 10, 20, 50, 100]	n_estimators: 100<br>max_depth: 15<br>min_samples_split: 2<br>min_samples_leaf: 1<br>bootstrap: False<br>max_leaf_nodes: None	n_estimators: 100<br>max_depth: 15<br>min_samples_split: 2<br>min_samples_leaf: 1<br>bootstrap: False<br>max_leaf_nodes: None
KNN	n_neighbors' : [5,7,9,11,13,15]<br>weights: ['uniform','distance']<br>metric:['minkowski','euclidean', 'manhattan']	n_neighbors: 13<br>weights: distance<br>Metric: Manhattan	n_neighbors: 15<br>weights: distance<br>Metric: Manhattan
SVM	C: [1, 10, 100]<br>gamma: [1,0.1,0.01,0.001]<br>kernel: ['rbf']	C: 1<br>gamma: 0.1<br>kernel: rbf	C: 100<br>gamma: 1<br>kernel: rbf
NB	naive Bayes has almost no hyperparameters to tune, so it usually generalizes well		
WLR	solvers = ['newton-cg', 'liblinear']<br>penalty = ['l1', 'l2']<br>c_values = [100, 10, 1.0, 0.1, 0.01]	solver: liblinear<br>penalty: l2<br>C: 0.001	solver: liblinear<br>penalty: l2<br>C: 0.1
WRF	n_estimators: [50, 100, 150, 200, 300]<br>max_depth: [None, 5, 10, 15, 20, 25, 30]<br>min_samples_split: [2, 5, 10]<br>min_samples_leaf': [1, 2, 4]<br>bootstrap: [True, False]<br>max_features: [auto, sqrt, log2]<br>max_leaf_nodes: [None, 5, 10, 20, 50, 100]	n_estimators: 50<br>max_depth: None<br>min_samples_split: 2<br>min_samples_leaf: 1<br>bootstrap: True<br>max_leaf_nodes: None	n_estimators: 300<br>max_depth: 15<br>min_samples_split: 2<br>min_samples_leaf: 1<br>bootstrap: True<br>max_leaf_nodes: None

Multimedia Appendix 2. Confusion Matrices

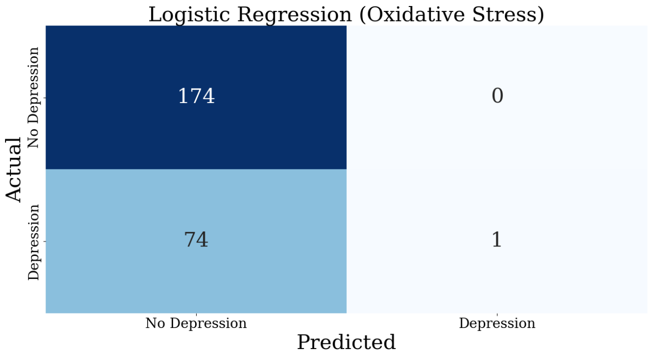

Figure 1: Confusion matrix of LR with oxidative stress biomarkers as the main features.

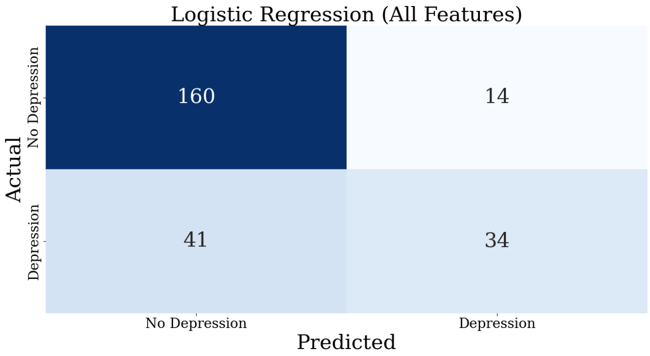

Figure 2: Confusion matrix of LR with all features.

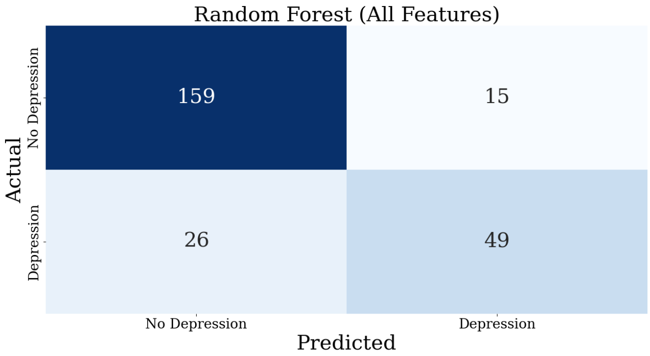

Figure 3: Confusion matrix of RF with all features.

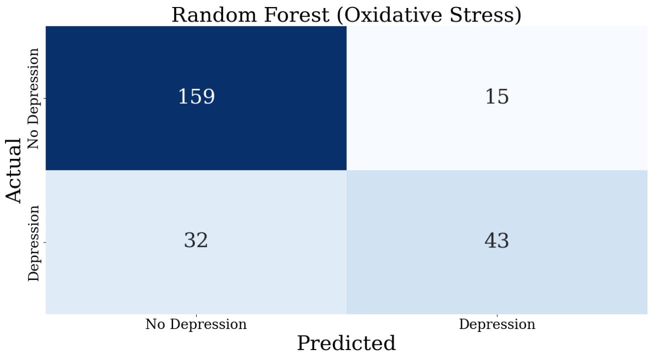

Figure 4: Confusion matrix of RF with oxidative stress biomarkers as the main features.

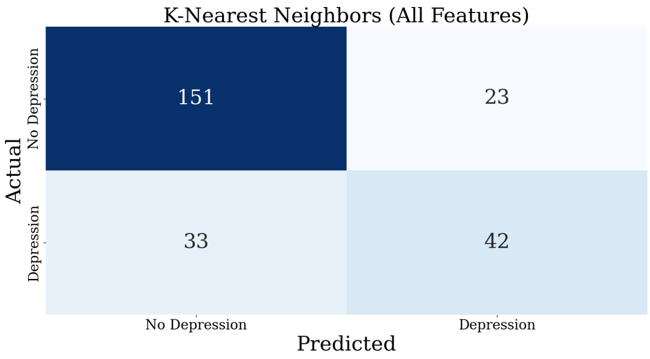

Figure 5: Confusion matrix of KNN with all features.

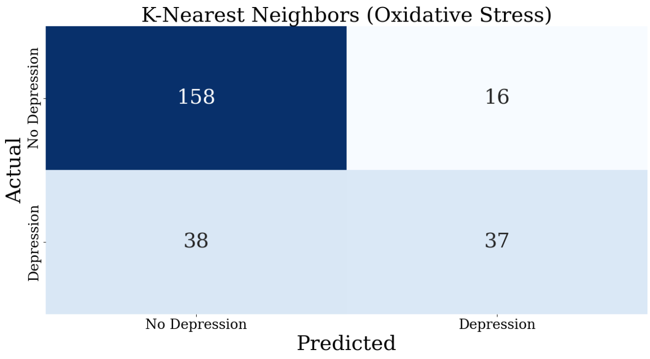

Figure 6: Confusion matrix of KNN with oxidative stress biomarkers as the main features.

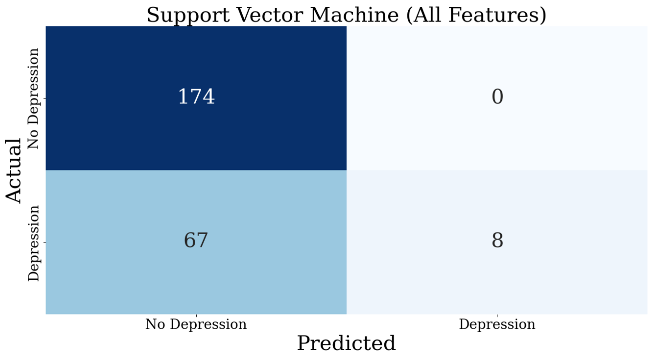

Figure 7: Confusion matrix of SVM with all features.

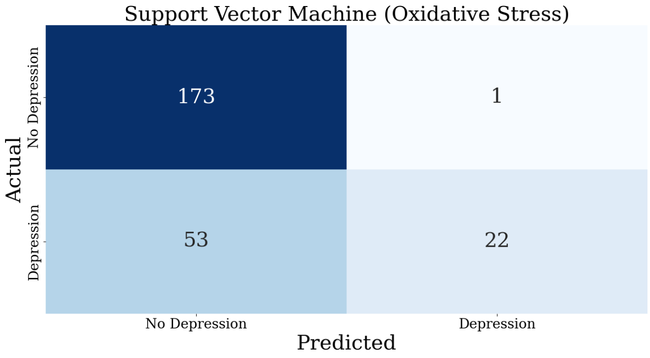

Figure 8: Confusion matrix of SVM with oxidative stress biomarkers as the main features.

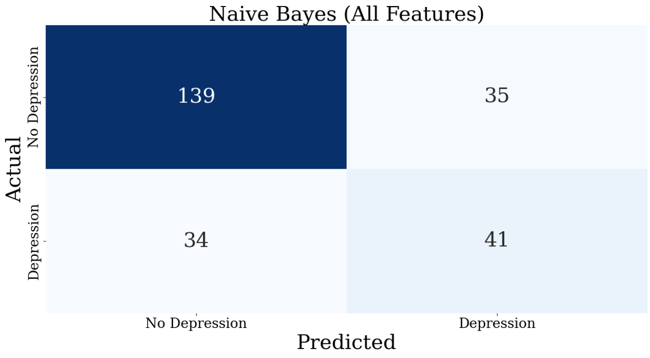

Figure 9: Confusion matrix of NB with all features.

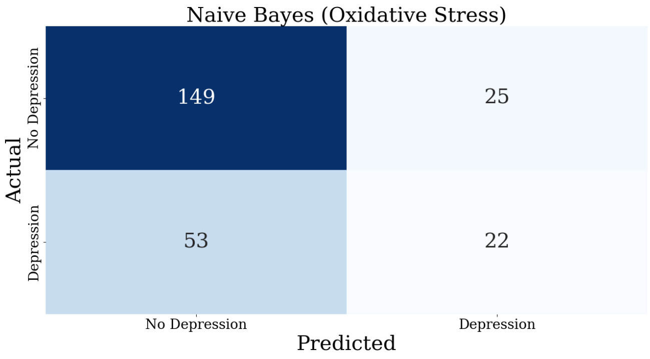

Figure 10: Confusion matrix of NB with oxidative stress biomarkers as the main features.

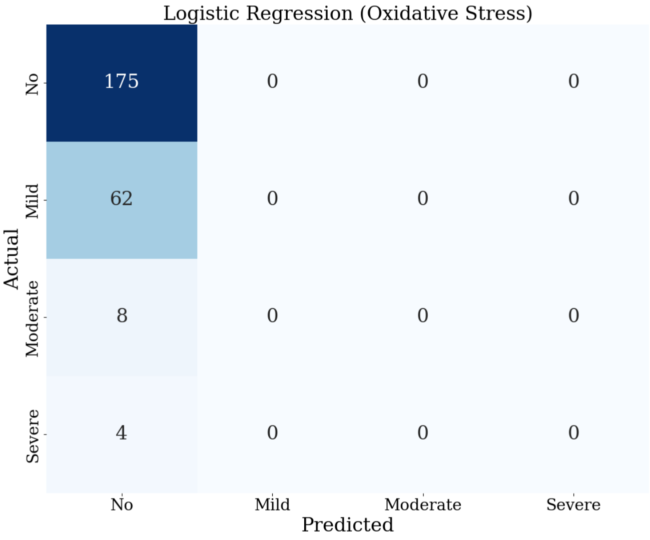

Figure 11: Confusion matrix of LR with oxidative stress biomarkers as the main features.

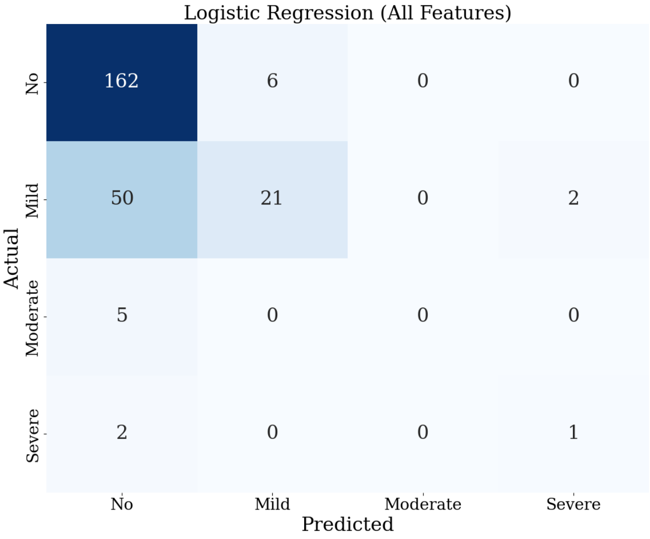

Figure 12: Confusion matrix of LR with all features.

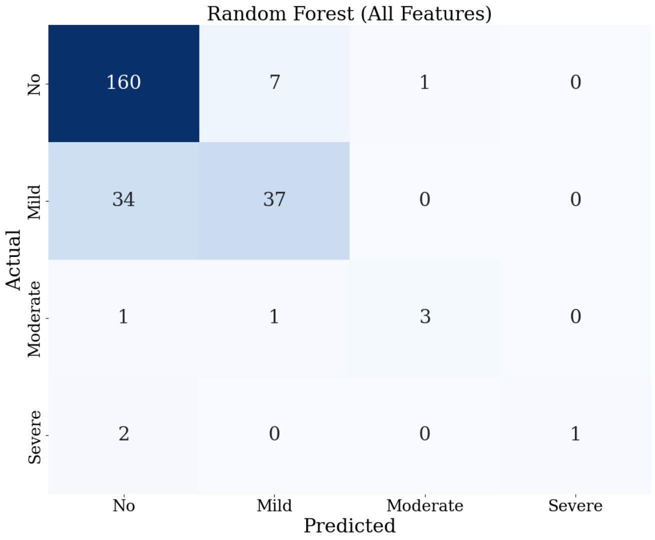

Figure 13: Confusion matrix of RF with all features.

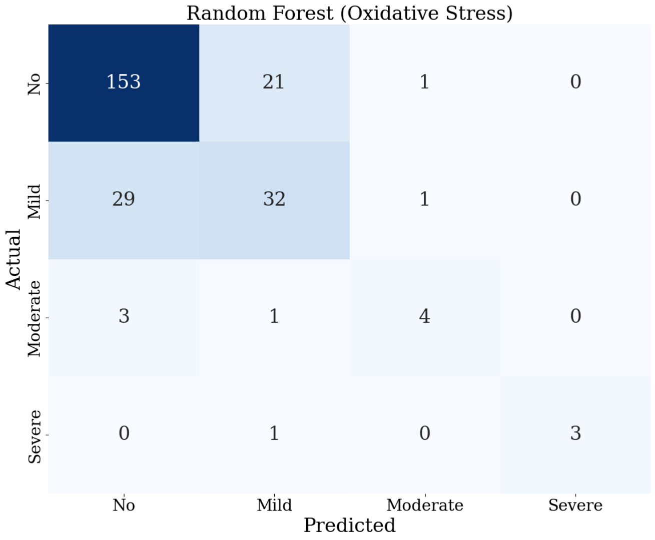

Figure 14: Confusion matrix of RF with oxidative stress biomarkers as the main features.

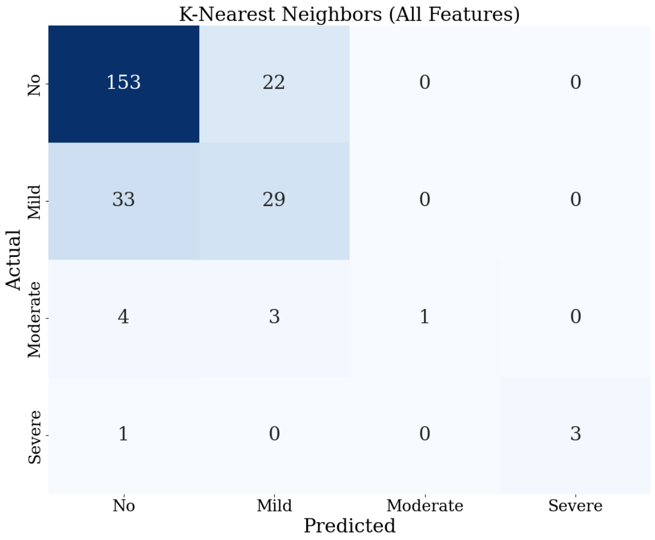

Figure 15: Confusion matrix of KNN with all features.

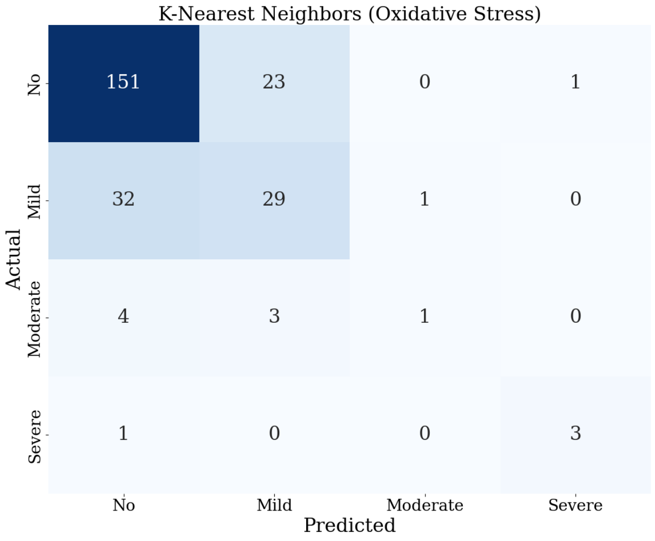

Figure 16: Confusion matrix of KNN with oxidative stress biomarkers as the main features.

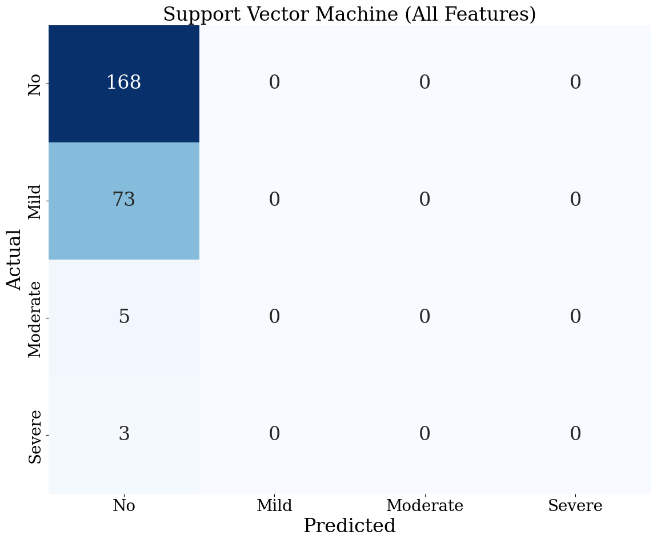

Figure 17: Confusion matrix of SVM with all features.

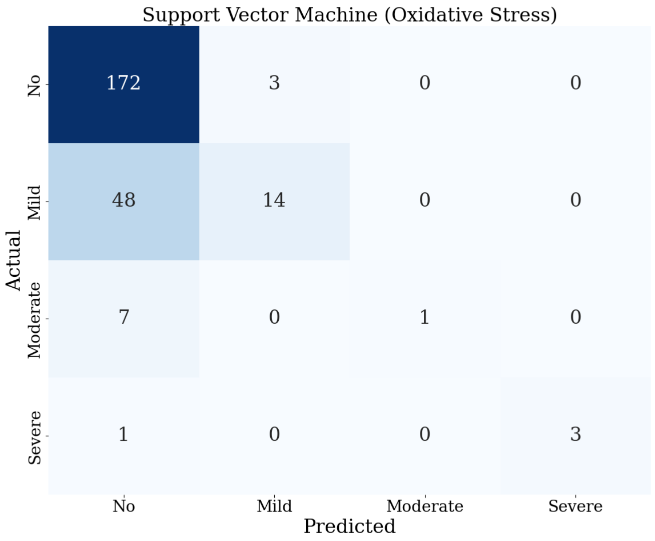

Figure 18: Confusion matrix of SVM with oxidative stress biomarkers as the main features.

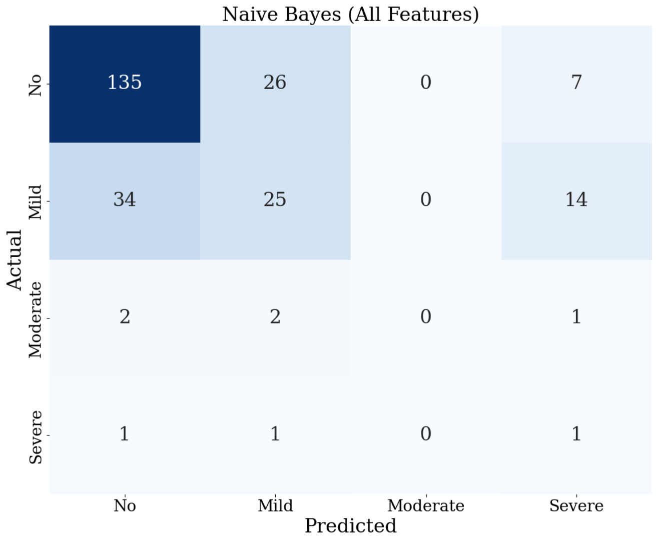

Figure 19: Confusion matrix of NB with all features.

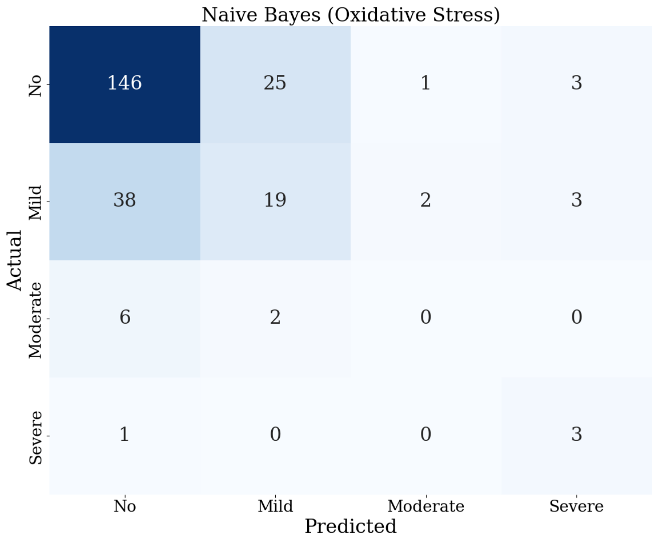

Figure 20: Confusion matrix of NB with oxidative stress biomarkers as the main features.

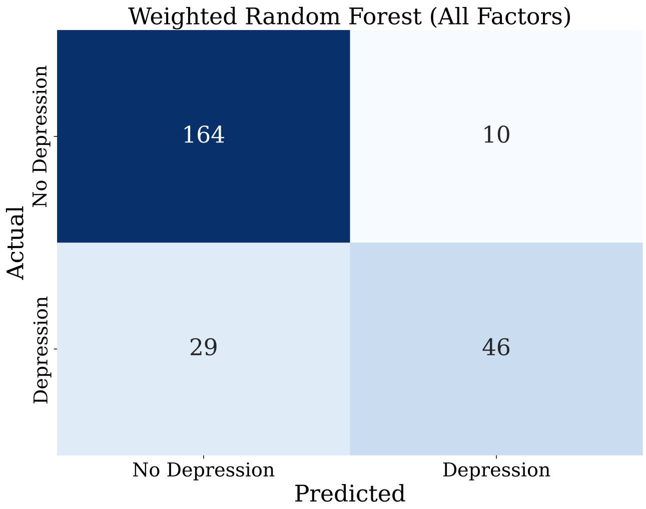

Figure 21: Confusion matrix of weighted RF with all features.

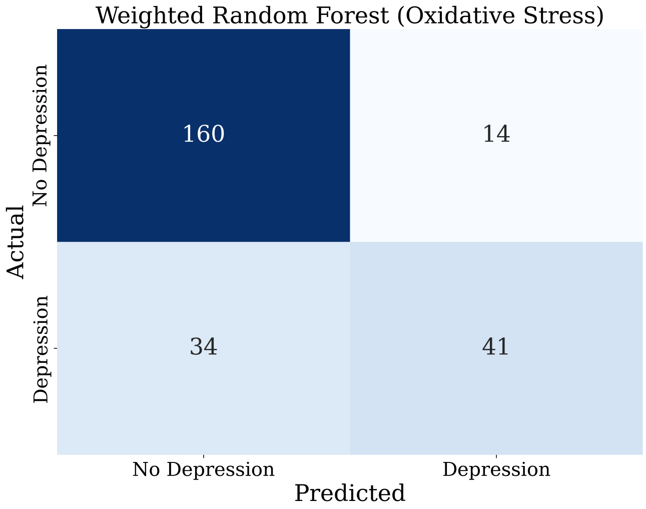

Figure 22: Confusion matrix of weighted RF with oxidative stress biomarkers as the main features.

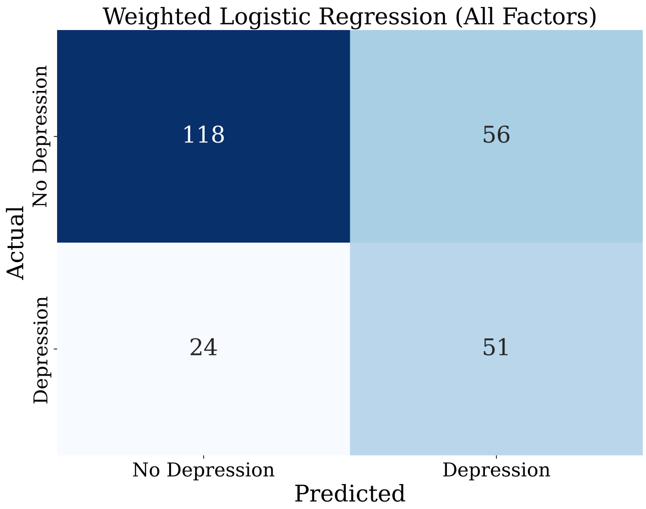

Figure 23: Confusion matrix of weighted LR with all features.

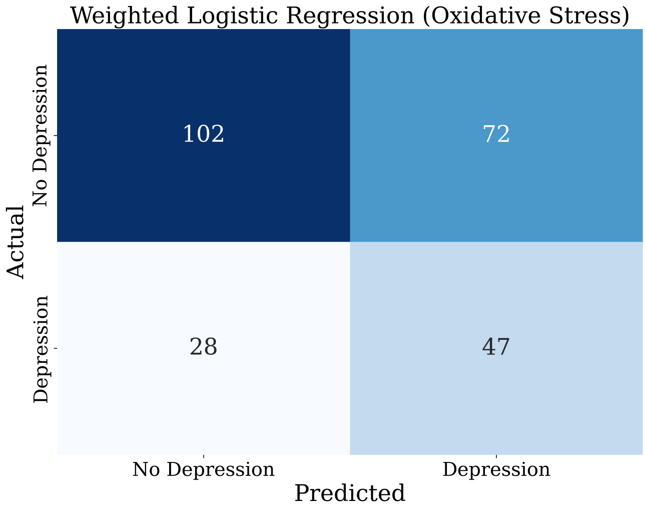

Figure 24: Confusion matrix of weighted LR with oxidative stress biomarkers as the main features.

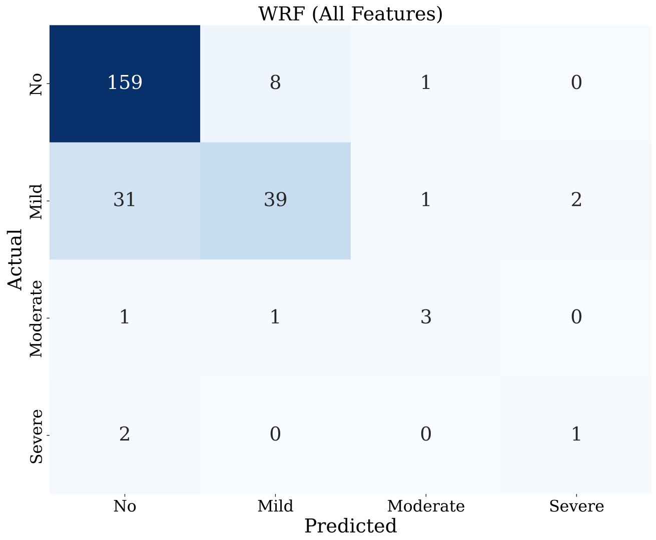

Figure 25: Confusion matrix of weighted RF with all features.

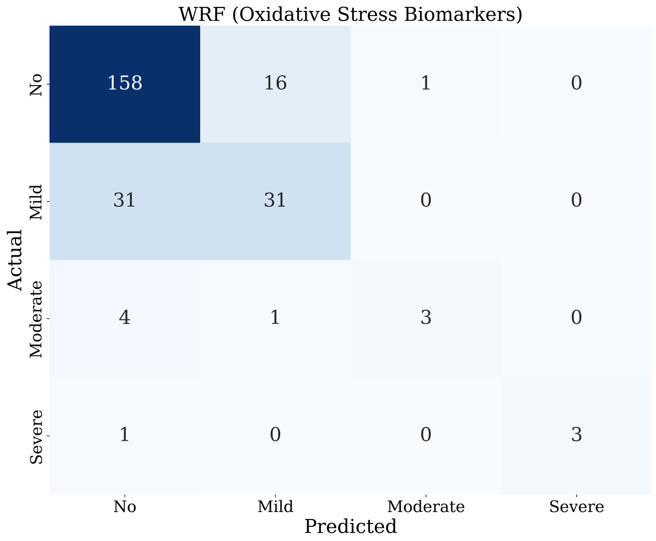

Figure 26: Confusion matrix of weighted RF with oxidative stress biomarkers as the main features.

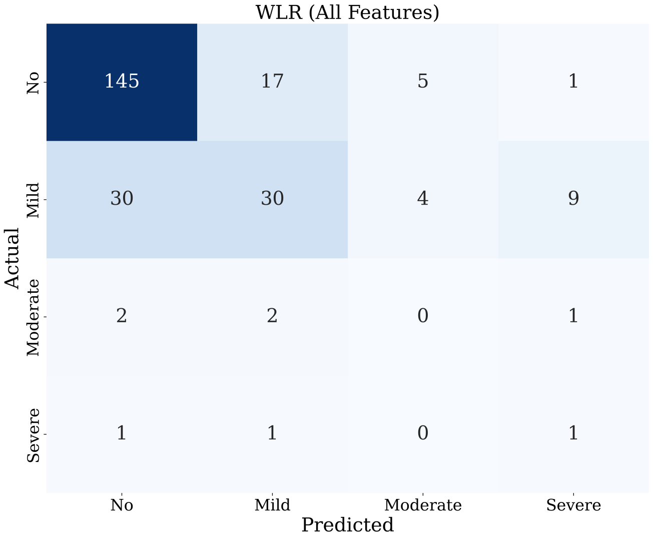

Figure 27: Confusion matrix of weighted LR with all features.

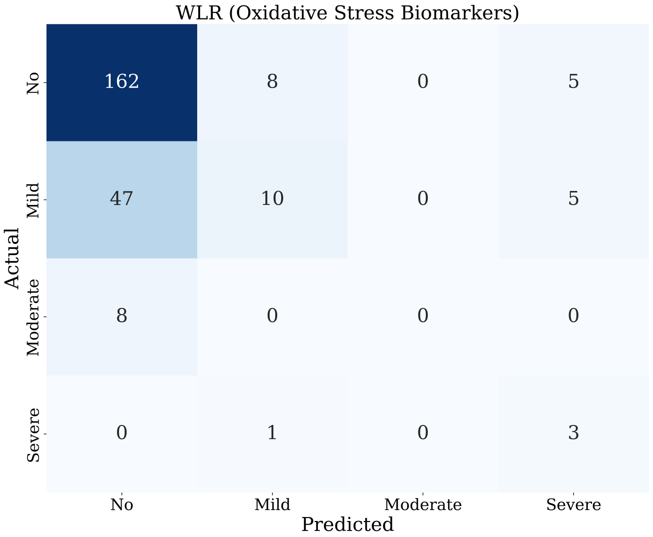

Figure 28: Confusion matrix of weighted LR with oxidative stress biomarkers as the main features.
